# *tailfindr*: alignment-free poly(A) length measurement for Oxford Nanopore RNA and DNA sequencing

**DOI:** 10.1261/rna.071332.119

**Published:** 2019-10

**Authors:** Maximilian Krause, Adnan M. Niazi, Kornel Labun, Yamila N. Torres Cleuren, Florian S. Müller, Eivind Valen

**Affiliations:** 1 Computational Biology Unit, Department of Informatics, University of Bergen, 5008 Bergen, Norway; 2 Sars International Centre for Marine Molecular Biology, University of Bergen, 5008 Bergen, Norway

**Keywords:** poly(A) tail, nanopore sequencing, cDNA, R package

## Abstract

Polyadenylation at the 3′-end is a major regulator of messenger RNA and its length is known to affect nuclear export, stability, and translation, among others. Only recently have strategies emerged that allow for genome-wide poly(A) length assessment. These methods identify genes connected to poly(A) tail measurements indirectly by short-read alignment to genetic 3′-ends. Concurrently, Oxford Nanopore Technologies (ONT) established full-length isoform-specific RNA sequencing containing the entire poly(A) tail. However, assessing poly(A) length through base-calling has so far not been possible due to the inability to resolve long homopolymeric stretches in ONT sequencing. Here we present *tailfindr*, an R package to estimate poly(A) tail length on ONT long-read sequencing data. *tailfindr* operates on unaligned, base-called data. It measures poly(A) tail length from both native RNA and DNA sequencing, which makes poly(A) tail studies by full-length cDNA approaches possible for the first time. We assess *tailfindr*’s performance across different poly(A) lengths, demonstrating that *tailfindr* is a versatile tool providing poly(A) tail estimates across a wide range of sequencing conditions.

## INTRODUCTION

The poly(A) tail is a homopolymeric stretch of adenosines at the 3′-end of the majority of eukaryotic mRNAs. These tails are necessary for the nuclear export of mature mRNAs (Hector et al. 2002; Bear et al. 2003; Fuke and Ohno 2008) and influence mRNA stability and translation (Eckmann et al. 2011).

The poly(A) tail is generated directly after transcription by the nontemplated addition of adenosines to the mRNA 3′-end, a process catalyzed by nuclear Poly(A)-polymerases (for review, see Millevoi and Vagner 2010). The initial length of poly(A) tails generated by this process has been estimated to be around 250 nt in vitro (Darnell et al. 1971; Edmonds et al. 1971; Raabe et al. 1991, 1994). After nuclear export, poly(A) length is dynamically regulated by the interplay of 3′-to-5′ degradation through exoribonucleases, poly(A) tail stabilization via poly(A) tail binding proteins, and elongation by cytoplasmic Poly(A)-polymerases (Diez and Brawerman 1974; Clegg and Pikó 1982; Hake and Richter 1994; Mendez et al. 2000; Read et al. 2002). While it has been shown that the poly(A) tail has a regulatory role, it is still not fully understood whether a specific length allows for specific regulatory outcomes (Jalkanen et al. 2014). A minimal poly(A) tail is needed to prevent quick 3′-to-5′ exonuclease degradation (Ford et al. 1997), yet hyperadenylated RNAs are marked for fast RNA degradation in the nucleus (Bresson and Conrad 2013; Jalkanen et al. 2014). Besides regulating RNA degradation, poly(A) tail length has been shown to correlate with translation efficiency during embryonic development (Beilharz and Preiss 2007; Subtelny et al. 2014), possibly by favoring a closed-loop structure of the mRNA. However, recent studies using *C. elegans* have proposed that shorter poly(A) tails are more actively translated, while longer tails are refractory to translation (Lima et al. 2017).

To understand the regulatory role of poly(A) tails, it is crucial to be able to measure poly(A) tail length genome-wide with transcript isoform resolution. Up until recently, estimating poly(A) tail lengths was restricted to transcript-specific measurements that relied on PCR and/or on laborious northern blotting techniques (Nilsen 2015). These techniques suffer from low throughput, high workload and possible technical artifacts due to amplification (Hite et al. 1996; Murray and Schoenberg 2008; Hommelsheim et al. 2014). Only recently a set of studies implemented short-read sequencing strategies to study poly(A) tail length in a transcriptome-wide manner (Chang et al. 2014; Subtelny et al. 2014; Lim et al. 2016; Balagopal et al. 2017; Lima et al. 2017; Woo et al. 2018). While these studies allowed a thorough understanding of poly(A) tail lengths throughout the transcriptome for the first time, they are technically restricted to a specific size of poly(A) tails depending on sample enrichment and sequencing strategy. Additionally, most of these techniques rely on PCR amplification of the poly(A) tail region, which might lead to amplification artifacts that affect poly(A) length measurements as well as quantitative comparisons between long and short poly(A) tails (Hite et al. 1996; Murray and Schoenberg 2008; Hommelsheim et al. 2014). Finally, and more importantly, these techniques can only indirectly identify the transcript linked to the poly(A) by alignment of short sequences representing the RNA 3′-ends. Thus it is challenging and in many cases virtually impossible to assign poly(A) tail measurements to specific transcript isoforms.

Oxford Nanopore Technologies’ (ONT) native RNA sequencing strategy allows for the sequencing of full-length mRNA molecules without amplification artifacts (Jain et al. 2016). The standard library preparation protocol retains the full poly(A) tail in the molecule to be sequenced, making it possible to obtain isoform-specific poly(A) tail length estimates in a transcriptome-wide manner (Garalde et al. 2018). However, current base-callers do not perform well on long homopolymer RNA and DNA stretches, resulting in the length of poly(A) tails not being accurately reported (Rang et al. 2018).

Here we present *tailfindr*, an R tool that estimates poly(A) tail length from individual reads directly from ONT FAST5 raw data. *tailfindr* is able to estimate poly(A) tails from both RNA and DNA reads, including DNA reverse-complement reads containing poly(T) stretches. *tailfindr* uses the raw data without prior alignment as input, and estimates the length based on normalization with the read-specific nucleotide translocation rate. We validate the performance of *tailfindr* on a set of RNA and DNA molecules with defined poly(A) tail lengths. *tailfindr* operates on the output of widely used as well as the most recent ONT base-calling applications (flip-flop model).

## RESULTS

### *tailfindr* estimates poly(A) tail length from base-called ONT native RNA sequencing

**FIGURE 1.**
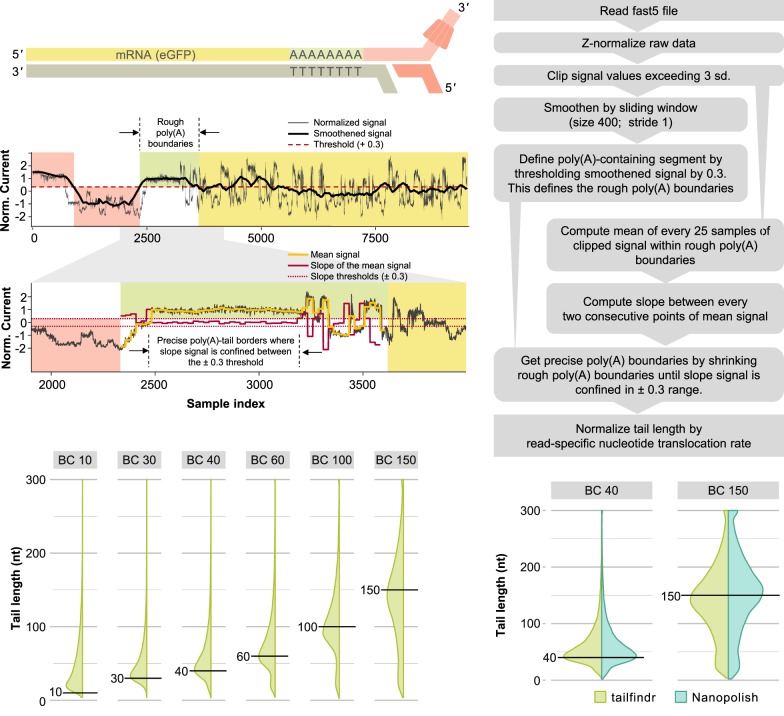
Workflow and performance of *tailfindr* on ONT RNA data. (*A*) Schematic representation of Oxford Nanopore RNA sequencing. The motor protein (red) is attached to the native RNA molecule (yellow) at the 3′-end by T4 DNA ligation via a double-stranded adapter (light red) with oligo-T overhang. The motor protein thus feeds the RNA strand to the pore from 3′ to 5′. (*B*) Representative signal tracks from eGFP-RNA sequencing. *Upper* panel shows normalized signal data calculated by *z*-normalization through *tailfindr* (gray, workflow box 3) with smoothened signal track (black, workflow box 4). Red background indicates ONT adapter signal and green background represents rough borders of poly(A) signal as identified by thresholding (workflow box 5), whereas yellow background highlights signal corresponding to potential RNA sequence. *Lower* panel shows zoom on potential poly(A) region with signal track for the mean of clipped, normalized raw data (yellow, workflow box 6) and slope of the mean signal track (red, workflow box 7), which are used to refine poly(A) boundaries (dashed *vertical* lines, workflow box 8). (*C*) Schematic workflow of data processing by the *tailfindr* algorithm for ONT native RNA sequencing data leading to signal tracks shown in *B* and ultimately poly(A) estimation. (*D*) Vertical density plots of poly(A) length estimation on in vitro transcribed eGFP-RNA molecules with known poly(A) tail length (from *left* to *right*: 10, 30, 40, 60, 100, and 150 nt labeled as BC10, BC30, BC40, BC60, BC100, and BC150, respectively). *Horizontal* black lines demarcate expected poly(A) length for individual barcodes. Poly(A) estimates exceeding 300 nt were set to 300 prior to plotting. (*E*) Vertical density plots of poly(A) length estimation from *tailfindr* (light green) and Nanopolish (turquoise) on in vitro transcribed eGFP-RNA with poly(A) length of 40 or 150 nt (labeled as BC40 and BC150, respectively). Poly(A) estimates exceeding 300 nt were set to 300 prior to plotting. Comparison of all known poly(A) lengths can be found in Supplemental Figure S2B.

Oxford Nanopore Technologies (ONT) sequencing allows for the sequencing of full-length native RNA molecules containing the entire poly(A) tail by ligation of a double-stranded DNA adapter to the 3′-end of each RNA molecule (Fig. 1A; Garalde et al. 2018). Indeed, long stretches of monotonous low-variance raw signal corresponding to poly(A) tails can be observed at the beginning of most reads (Fig. 1B). However, since base-calling relies on fluctuations of the raw signal, these low-variance sections are poorly decoded into the correct nucleobase sequence (Rang et al. 2018).

To identify the region corresponding to the expected poly(A) tail, we apply thresholding to normalized raw data, refine the boundaries of possible poly(A) stretches based on raw signal slope, and normalize by the read-specific nucleotide translocation rate (Fig. 1C, for details see Materials and Methods). *tailfindr* provides the user with a tabular output containing the unique read-ID, the estimated poly(A) tail length and all factors extracted from the raw data that are needed to calculate the poly(A) tail estimate (Supplemental Fig. S4A). This allows for custom filtering of the acquired poly(A) measurements by the user. Optionally, *tailfindr* allows the user to generate read-specific plots displaying the raw data and all signal derivatives generated in the process to estimate poly(A) tail length (Supplemental Fig. S4B). To test the performance of our algorithm, we pooled six barcoded in vitro transcribed eGFP RNA samples with different poly(A) tail lengths (10, 30, 40, 60, 100, and 150 nt) and sequenced the pooled samples with ONT's native RNA sequencing kit in two replicates. Because the barcodes that define molecules with specific poly(A) length are located at the 5′-end of the eGFP RNA, only reads that cover the full RNA molecule from 5′-end to 3′-end were considered for the analysis. After barcode demultiplexing, the estimated poly(A) tail lengths for each length group overall match the expected poly(A) tail length, with the exception of eGFP with a poly(A) tail of 10 nt (Fig. 1D). While the molecules with an expected poly(A) length of 10 nt were measured with a mode of 21, the mode of poly(A) measurements of all other barcoded RNA molecules matches well with the expected poly(A) lengths (30 nt: 33; 40 nt: 41; 60 nt: 59; 100 nt: 91; 150 nt: 136). However, even though the majority of sequences show the expected poly(A) tail length, the standard deviation of poly(A) tail measurements is relatively high (coefficient of variation between 45% and 79%, see Table 1). This is not a result of poor poly(A) tail boundary assignment, as poly(A) tail end coordinates defined by *tailfindr* match with coordinates from alignment of the expected adjacent eGFP sequence with a precision of around 2 nt (Supplemental Fig. S6; Supplemental Discussion). Both the high accuracy of poly(A) length estimation as well as the variation around the average is consistent across replicates (Supplemental Fig. S1A). Furthermore, the estimates are robust across different sequencing conditions, as a third replicate performed with the new Library preparation kit (SQK-RNA002) and omitting the optional Reverse Transcription reaction resulted in similar poly(A) length measurements (Supplemental Fig. S1B). Thus, while the poly(A) estimation suffers from significant variation, the length of most barcoded molecules can be successfully estimated by the use of *tailfindr* on ONT RNA sequencing.

**TABLE 1.**
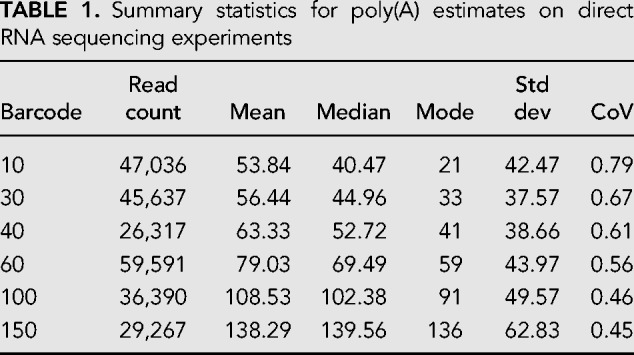
Summary statistics for poly(A) estimates on direct RNA sequencing experiments

While this study was in progress, another tool estimating poly(A) tail lengths from ONT RNA data was developed (Workman et al. 2018). Instead of estimating poly(A) tails from base-called data directly, this tool requires read alignment information for the definition of the poly(A) tail segment. To compare whether our algorithm results in similar performance, we measured poly(A) tail lengths from Nanopolish and *tailfindr* on different barcoded eGFP molecules. Our analysis showed that both tools matched well in their estimated poly(A) tail lengths, as exemplified in Figure 1E for 40 and 100 nt poly(A) tail length (full comparison including analyses on published data set by Workman et al. 2018 in Supplemental Fig. S2). However, while both tools agreed in the majority of cases on the definition of poly(A) segments, we routinely observed slightly higher estimates from Nanopolish which can be attributed to differences in normalization (Supplemental Fig. S3A,B). In conclusion, *tailfindr* accurately defines poly(A) tail segments in ONT native RNA sequencing data and provides similar estimates to Nanopolish while only using base-called data files as input.

### Poly(A) and poly(T) tail length can be estimated from ONT DNA sequencing data

**FIGURE 2.**
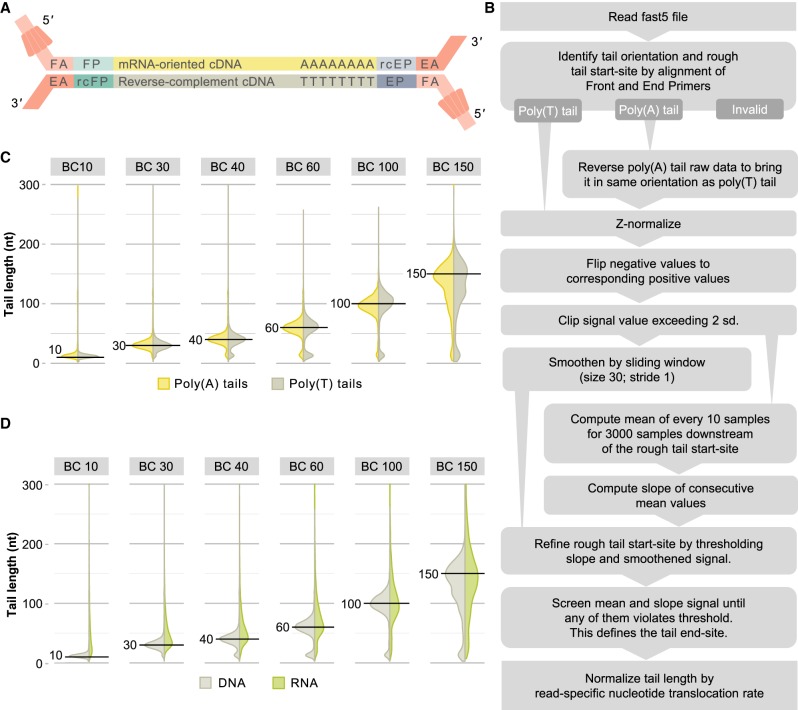
Workflow and performance of *tailfindr* on ONT DNA sequencing data. (*A*) Schematic representation of Oxford Nanopore DNA sequencing. In cDNA approaches, amplification is ensured by oligo-dT-aided anchoring of the end primer (EP, blue) and addition of front primer sequence (FP, light green) by template switching during reverse transcription. The motor protein (red) is attached to the double-stranded DNA molecules at both ends by T4 DNA ligation. The front adapter (FA) bears the motor protein, while the end adapter (EA) is a short complementary oligo that will ultimately appear at the 3′-end of resulting sequences. Both DNA strands are sequenced from 5′ to 3′. Thus, oligo-dT stretches will be present at the beginning of raw data, while oligo-dA stretches appear at the end. (*B*) Schematic workflow for ONT DNA sequencing data processing by the *tailfindr* algorithm. (*C*) Vertical density plot of poly(A) (yellow) and poly(T) (gray) length estimates on PCR-amplified eGFP coding sequence with known poly(A) length. *Horizontal* black lines demarcate expected poly(A) length for individual barcodes (from *left* to *right*: 10, 30, 40, 60, 100, and 150 nt labeled as BC10, BC30, BC40, BC60, BC100, BC150, respectively). (*D*) Vertical density plot of poly(A)/(T) length estimates on DNA sequences (gray) and poly(A) length estimates on RNA (light green) (from *left* to *right*: 10, 30, 40, 60, 100, and 150 nt labeled as BC10, BC30, BC40, BC60, BC100, BC150, respectively).

ONT native RNA sequencing is lower in both quantity and quality compared to cDNA sequencing approaches and relies on large amounts of starting material [500 ng of poly(A)-selected RNA, Oxford Nanopore Technologies 2018a, 2019]. Therefore, cDNA sequencing approaches that retain the full-length poly(A) tail would enable studies where material is scarce as well as increase statistical power of poly(A) tail estimates. We thus aimed to expand *tailfindr* to operate on ONT DNA sequencing approaches as well. Since standard cDNA approaches result in double-stranded DNA, both poly(A) as well as poly(T) stretches are present in ONT sequencing reads. During cDNA sequencing both of these strands are threaded through the pore separately from 5′ to 3′ (Fig. 2A). Indeed we observe homogenous stretches of raw signal both at the beginning [poly(T) tail] as well as at the end [poly(A) tail] of individual raw read sequences [example for poly(T)-containing read in Supplemental Fig. S5B].

We extended our algorithm to accommodate ONT DNA sequencing data output (Fig. 2B). Running the algorithm provides the user with a tabular output of tail length measurements as well as optional raw data plots (Supplemental Fig. S5). We account for the double-stranded nature of DNA and define the read type [poly(A)- or poly(T)-containing] by making use of known sequence motifs in Nanopore adapters (details in Materials and Methods). We tested the performance of the DNA-specific *tailfindr* algorithm on PCR products of eGFP coding sequence with known poly(A)/(T) length in two replicates, similar to the spike-ins generated for native RNA sequencing. As shown in Figure 2C, the DNA-specific *tailfindr* approach resulted in estimated poly(A) and poly(T) lengths close to the expected length for barcoded molecules [mode of distribution for 10 nt: 10; 30 nt: 29; 40 nt: 39; 60 nt: 59; 100 nt: 97 for poly(A) and 110 for poly(T); 150 nt: 148 for poly(A) and 155 for poly(T)]. These estimates were consistent across replicates from different Library preparation kits (Supplemental Fig. S7) and the poly(A)/(T) end coordinates matched with coordinates of the alignment of adjacent eGFP sequence (Supplemental Fig. S6). For all poly(A)/(T) tail lengths bigger than 10 nt, a small subpopulation of reads with shorter estimated tails could be observed, possibly due to amplification artifacts that connect barcoded eGFP sequence with wrong poly(A) tail lengths (see Supplemental Discussion; Supplemental Fig. S8).

Next we compared poly(A)-length estimates from DNA and native RNA sequencing. We observed that DNA sequencing results in significantly more precise estimation of poly(A) tail length, mainly due to fewer outliers toward longer poly(A) tail lengths (Fig. 2D). Especially the shortest poly(A) tail length (10 nt) could be estimated more correctly with DNA sequencing [mode of poly(A) length estimation 10 in DNA vs. 22 in RNA sequencing]. On other poly(A) lengths, the mode of poly(A) estimation does not differ dramatically, but the precision is significantly higher for DNA sequencing (coefficient of variation between 33% and 50% in DNA sequencing, Table 2). In summary, *tailfindr* is able to estimate poly(A) and poly(T) tail size from ONT DNA sequencing with significantly higher precision compared to ONT RNA sequencing estimates.

**TABLE 2.**
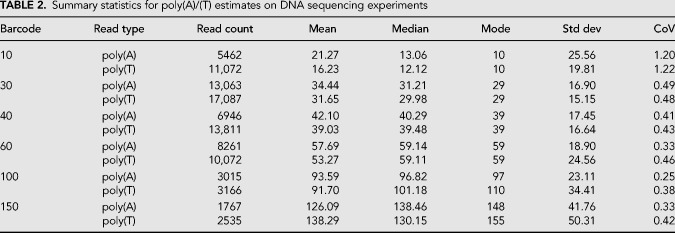
Summary statistics for poly(A)/(T) estimates on DNA sequencing experiments

### *tailfindr* is compatible with flip-flop model base-calling

While this manuscript was in preparation, ONT released a new DNA base-calling strategy based on flip-flop models. Flip-flop model base-calling screens the raw signal by comparing probabilities to either stay in the same nucleotide state or change to a new state. Additionally, the raw data is read by averaging over two sample points only, as opposed to averaging over five sample points in standard model base-calling. These improvements have been shown to result in higher quality base-calling, and more importantly to increase the base-call fidelity over homopolymer sequences (Oxford Nanopore Technologies 2018b). So far, flip-flop model base-calling is only available for ONT DNA sequencing data.

**FIGURE 3.**
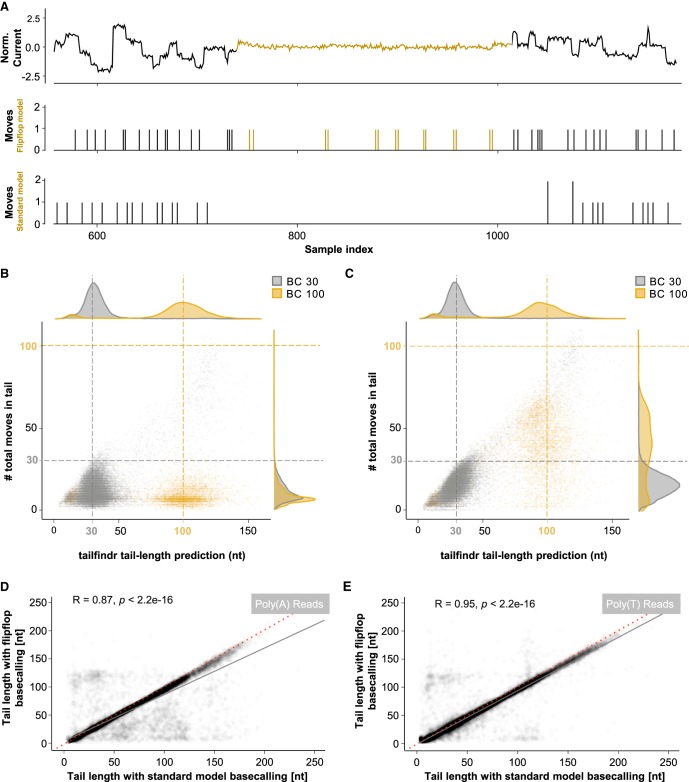
Differences in poly(A) tail estimation for standard and flip-flop model base-calling. (*A*) Representative raw data squiggle of PCR-amplified eGFP coding sequence over the identified poly(A) tail region (colored yellow) with associated moves (shifts in raw data representing possible nucleotide translocations) in both flip-flop (*middle* panel) and standard model base-calling (*bottom* panel). Flip-flop model base-calling detects moves with higher resolution, and calls more moves, especially in the poly(A) tail region (yellow). (*B*,*C*) Scatter plot of estimated poly(A)/(T) tail length (*x*-axis) and moves detected with standard (*B*) or flip-flop model base-calling (*C*) on PCR-amplified eGFP coding sequence with poly(A) length of 30 nt (gray) and 100 nt (yellow). Colored dashed lines indicate expected poly(A) length. (*D*,*E*) Scatter plot of poly(A) (*D*) or poly(T) (*E*) tail length estimated from PCR-amplified eGFP coding sequence with different poly(A) tail lengths that were base-called either with standard (*x*-axis) or flip-flop models (*y*-axis). (R, p by Pearson correlation). Red dashed line indicates *x* = *y*; gray line indicates linear fit.

We implemented changes in *tailfindr* to account for the updates in flip-flop model raw data output. As expected, flip-flop model base-calling detects more nucleotide translocations (called “moves”) over poly(A) stretches when compared to standard model base-calling (Fig. 3A, yellow highlights). To test whether the detected moves agree with expected poly(A)/(T) length, we plotted the moves from either standard model base-calling (Fig. 3B) or flip-flop model base-calling (Fig. 3C) on eGFP-PCR products with 30 or 100 nt poly(A)/(T) tail length. While flip-flop model base-calling resulted in significantly more detected moves over poly(A)/(T) tail sections compared to standard model base-calling, the number of moves still severely underestimates existing poly(A)/(T) lengths. Thus even with improved homopolymer base-call fidelity, external tools are needed to correctly measure poly(A) tail lengths. We used *tailfindr* to compare poly(A) and poly(T) tail measurements from the same sequencing reads base-called either with flip-flop or standard models, and could show that the estimated poly(A)/(T) tail length is highly correlated between the two base-calling approaches [*R* = 0.93 for poly(A); *R* = 0.97 for poly(T); Fig. 3D,E]. We thus conclude that *tailfindr* operates on both standard and the most recent flip-flop model base-calling, and provides accurate poly(A)/(T) length estimates for ONT DNA sequencing approaches.

## DISCUSSION

Polyadenylation at the 3′-end is understood to be a major regulator of mRNA (Hector et al. 2002; Bear et al. 2003; Fuke and Ohno 2008; Eckmann et al. 2011). While the poly(A) length of mRNAs has been under investigation since the 1970s (Brawerman 1973; Morrison et al. 1973; Groner et al. 1974; Merkel et al. 1976), transcriptome-wide analysis of poly(A) tail lengths have only recently emerged. The advent of Oxford Nanopore Technologies (ONT) native RNA sequencing technology now allows direct sequencing of full-length mRNA molecules, which intrinsically contain their full poly(A) tail, unbiased by potential amplification artifacts (Jain et al. 2016). However, even the most recent updates in base-calling tools do not perform well over long homopolymeric sequence stretches (Oxford Nanopore Technologies 2018b; Rang et al. 2018).

In this work we present *tailfindr*, a versatile R tool that allows estimation of poly(A) tail lengths from base-called ONT long-read sequencing data from both native RNA and DNA sequencing approaches. *tailfindr* operates on data from all current and previous ONT base-calling strategies that produce an events/move table in the resulting FAST5 files. We show that *tailfindr* is able to detect the poly(A) tail boundaries of in vitro transcribed eGFP RNA molecules and estimate their lengths based on read-specific raw data normalization. For molecules with known poly(A) tails from 30 nt up to 150 nt the estimates match well with the expected lengths (Fig. 1D), however the shortest poly(A) tail (10 nt) was estimated to have longer tails than expected. We believe that this bias can be explained by sample contamination of this RNA molecule during preparations, or by inefficient oligo-dT sequencing adapter ligation to poly(A) tail stretches at or below 10 nt. Consistent with the latter explanation we observed that the barcoded 10 nt RNA molecule was underrepresented in the RNA sequencing libraries compared to input quantities (Table 3). Overall, *tailfindr* correctly estimates poly(A) tail lengths of in vitro transcribed RNA over a wide range of lengths.

**TABLE 3.**
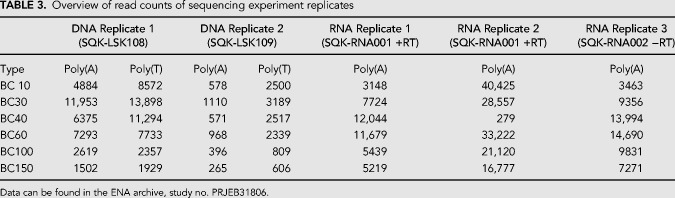
Overview of read counts of sequencing experiment replicates

We further show that *tailfindr* poly(A) tail estimates agree closely with a recently developed tool that relies on the prior mapping of the data (Workman et al. 2018). While poly(A) tail boundaries in the raw signal are found to be essentially the same with the two different approaches (Supplemental Fig. S2C,D), the final calculated poly(A) tail lengths differ slightly (Supplemental Fig. S2A). Specifically, *tailfindr* estimates short poly(A) stretches slightly longer than Nanopolish, while long poly(A) stretches result in shorter estimates in *tailfindr.* These differences can be explained by a different calculation of the average nucleotide translocation rate (Supplemental Fig. S2B) which is used to normalize raw poly(A) tail measurements. Nanopolish normalizes by calculating the read-specific median of the samples per nucleotide after removing 5% of the translocation rate outliers. We observed that this normalization is resulting in correct poly(A) estimation in RNA, but not DNA sequencing approaches (further discussed in Supplemental Discussion). Instead, we normalize by the read-specific geometric mean of samples per nucleotide without a specific arbitrary outlier threshold. Another difference between the tools is that *tailfindr* does not need any sequence data preprocessing, as it only requires base-called FAST5 files with an events table as input. This allows for poly(A) tail studies independent of any other tool than the essential base-caller, which would allow for an integration of the *tailfindr* algorithm into the base-calling procedure. This in turn makes it possible to assign poly(A) tail lengths to individual reads in parallel to the sequencing procedure, making live poly(A) tail analysis feasible.

In comparison to recent short-read sequencing-based strategies to measure poly(A) tails, methods using ONT sequencing are currently less precise. Short-read sequencing approaches promise poly(A) measurements with just a few bases of deviation due to cyclic incorporation of nucleotides and integration of the fluorescence signal of multiple molecules toward one single base-call (Chang et al. 2014; Lim et al. 2016; Balagopal et al. 2017; Lima et al. 2017; Woo et al. 2018). In contrast, ONT long-read sequencing measures individual single-stranded molecules, and single nucleotide changes are detected based on subtle changes in measured current levels. More importantly, the raw signal for ONT sequencing does not change over a homopolymeric region, making single-event detection almost impossible. Thus, ONT poly(A) length estimation relies on normalization of variable data taken from single-molecule measurements. Most of the variation observed in *tailfindr* poly(A) estimation thus comes from the sequencing process. However, the sequencing chemistry as well as the properties of the motor protein is under constant development. It is thus conceivable that in the near future an increase in speed and robustness of translocation rates can be observed, which will have a positive impact on poly(A) tail estimation (Oxford Nanopore Technologies 2018b). Updates in both sequencing chemistry as well as base-calling strategies can dramatically change the appearance and data obtained by base-calling. This is exemplified by the differences observed for base-calling the same data with standard and flip-flop models (Fig. 3; Supplemental Discussion). While these changes could in theory render the described algorithms imprecise or worst nonfunctional, future changes are more likely to reduce the variability and increase the precision of the nucleotide translocation rate. This would address the methods’ current weakness and result in increased accuracy for poly(A) tail estimation using our *tailfindr* algorithm.

While currently not as precise in measuring poly(A) tails, ONT long-read sequencing approaches have unique advantages over short-read sequencing approaches. First, ONT sequencing is intrinsically a single-molecule technique. Second, RNA sequencing approaches are amplification-free, avoiding the emergence of possible amplification artifacts. Third, since the native molecule is sequenced as it comes from the specimen, additional features of the RNA can be measured directly, as was shown for RNA modifications (Viehweger et al. 2018; Workman et al. 2018). Fourth, and most importantly, long-read sequencing allows direct assignment of transcript isoforms to single molecules without bioinformatics post-processing, making truly isoform-specific measurements of poly(A) tail lengths possible. Additionally, ONT sequencing allows to study features of 5′-end and 3′-end events of the same molecule in conjunction with the poly(A) tail length. Together, ONT sequencing in conjunction with *tailfindr* poly(A) estimation offers great potential to combine the study of poly(A) tail length and other RNA features with transcript-isoform specificity in one assay.

Beyond ONT RNA sequencing applications, *tailfindr* is the first tool to show that poly(A) tails can be measured in ONT DNA sequencing. For DNA sequencing approaches *tailfindr* handles the most up-to-date base-calling strategy using flip-flop model base-calling (Fig. 3), making *tailfindr* compatible with all recently produced data sets. Interestingly, poly(A) estimation from ONT DNA sequencing is far more precise compared to measurements of similar RNA molecules (Fig. 2D). This is likely explained by a faster and more robust translocation rate with less likelihood for stochastic stalling during sequencing.

*tailfindr* makes it possible to design specific cDNA library preparation protocols that retain the full poly(A) tail in ONT sequencing approaches. This strategy has recently been shown to allow further insights into poly(A) tail regulation based on PacBio long-read sequencing (Legnini et al. 2019). ONT cDNA sequencing has the advantage to yield approximately 10× more data per library preparation compared to native RNA sequencing, and due to amplification would allow sequencing experiments starting with minute RNA amounts as input (Oxford Nanopore Technologies 2018c, 2019). Additionally, we envision that future cDNA applications using Unique Molecular Identifiers (UMI) will make it possible to acquire multiple poly(A) tail measurements from each molecule, which would increase the fidelity of isoform-specific poly(A) tail measurements. Thus, using *tailfindr* with specific ONT cDNA applications offers new approaches to study the role of poly(A) tail lengths from scarce biological samples.

In conclusion, ONT RNA sequencing offers a new possibility to study poly(A) tail biology by directly associating poly(A) tail length with other RNA features in a transcript isoform-specific manner. *tailfindr* has proven successful in measuring the poly(A) tail of both RNA and DNA sequencing solely from base-called raw data, an approach that allows real-time analysis during ONT long-read sequencing. With the application of *tailfindr* for ONT DNA sequencing we allow future development of poly(A)-retaining cDNA sequencing assays that further increase the ability to study poly(A) tail lengths from limited material.

## MATERIALS AND METHODS

### Spike-in generation

To generate RNA with known poly(A) tail lengths, we used eGFP as a carrier RNA as it fulfills basic criteria for successful ONT RNA sequencing (especially minimal length requirement). The coding sequence of eGFP was amplified from pCS2+ −eGFP vector using High Fidelity Phusion MasterMix (ThermoFisher, #F-531L). The primers for the PCR included the SP6 promoter sequence and a barcode in the forward primer, as well as a homopolymer T stretch in the reverse primer (see Table 4). After gel purification of the desired PCR product, a second PCR was performed with a reverse primer that introduces a Bfo1 restriction site before the homopolymer T stretch (polyA Bfo1 rev, together with SP6 Bfo1 fw, Table 4). After gel purification and Phenol–chloroform extraction, the resulting PCR products were used for Nanopore DNA ligation sequencing (see below). For preparation of RNA spike-ins, the PCR products were digested with FastDigest Bfo1 (ThermoFisher, #FD2184) for 2 h and purified by Phenol–chloroform extraction. An amount of 100–300 ng of purified DNA was used for RNA in vitro transcription by the SP6 mMessage mMachine kit (ThermoFisher, #AM1340) following the manufacturer's procedures. The resulting RNA was purified using Zymo RNA Clean & Concentrator-5 columns (Zymo Research, #R1013).

**TABLE 4.**
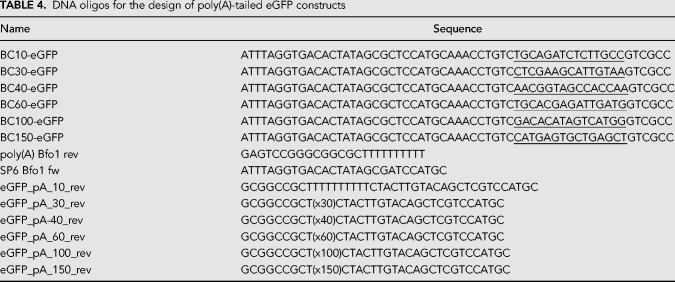
DNA oligos for the design of poly(A)-tailed eGFP constructs

### ONT long-read sequencing

Native RNA sequencing was performed on two replicates using the ONT kit SQK-RNA001 following the manufacturer's protocol. One additional replicate was performed using the kit SQK-RNA002 omitting the reverse transcription reaction described in the manufacturer's protocol. In brief, 500 ng of poly(A)-selected RNA was mixed with 100 ng of poly(A) spike-in RNA, or 500 ng poly(A) spike-in RNA was used alone. The RNA was ligated to ONT RT adapter (RTA) and used for reverse transcription with SuperScript II (ThermoFisher, #18064022; omitted for third replicate). Next, the proprietary sequencing adapter was ligated using T4 DNA ligase (NEB, #M0202M) and loaded onto ONT Sequencing Flow Cells (FLO-MIN106 R9.4.1). Sequencing was performed for 16–24 h using MinKNOW 2 software. All RNA purification steps were performed with RNAClean XP beads (Beckham Coulter, #A63987) with 15 min incubation intervals.

DNA sequencing was performed using the DNA Ligation Kits SQK-LSK108 and SQK-LSK109 on poly(A)-containing PCR products. In brief, 500 ng of pooled barcoded PCR products were end-prepped using the NEBNext Ultra II dA tailing module (NEB, #E7546S) and ligated to proprietary sequencing adapters using T4 DNA ligase (NEB, #M0202M). Purified libraries were sequenced on flow cells (FLO-MIN106 R9.4.1) for 24 h using MinKNOW 2.

### Sequencing data processing

RNA and DNA raw reads were base-called using Albacore v2.3.3. DNA raw reads were additionally base-called with Guppy v2.3.1 using the flip-flop model. Sequencing quality and general metrics were assessed using NanoPlot (v1.19.0, De Coster et al. 2018). Reads that passed the default albacore quality filter were mapped against the eGFP sequence using minimap2 (v2.14-r883) with default settings for ONT data mapping (-ax splice -uf -14 for RNA; -ax splice for DNA, Li 2018).

### Demultiplexing barcoded spike-ins

All alignments discussed in this manuscript, unless mentioned otherwise, were performed using Smith–Waterman local alignments with Biostrings (Pages et al. 2019) (match score 1; mismatch score -1; gap opening penalty 0; and gap extension penalty 1). The normalized alignment score was calculated by dividing the local alignment score by the length of the query sequence. If not otherwise mentioned, alignments with a normalized alignment score below 0.6 were discarded as unspecific.

Barcoded eGFP RNA reads with known poly(A) length were demultiplexed by locating the first 29 bases of eGFP sequence (see Table 5) within the first 250 bases of FASTA strings extracted from every FAST5 file. Next, the barcode was assigned by aligning the expected barcode sequences against the extracted read sequence preceding the eGFP alignment (see Table 5). The barcode with highest normalized alignment score (and above threshold of 0.6) was assigned to the read.

**TABLE 5.**
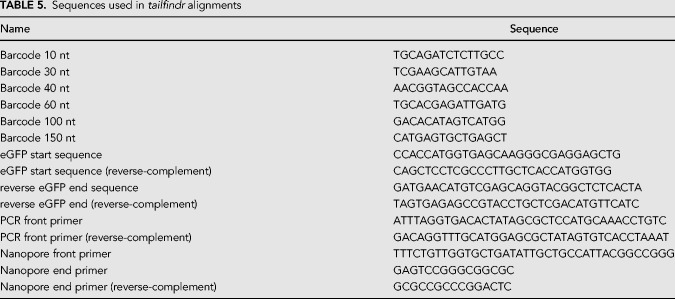
Sequences used in *tailfindr* alignments

To analyze barcoded eGFP DNA reads, the orientation of reads was investigated by aligning the first 29 bases of eGFP and its reverse-complement (Table 5) to the first 250 bases of FASTA strings extracted from each FAST5 file. A read was considered a poly(A)-containing read if the normalized alignment score of eGFP sequence was greater than both the normalized alignment score of the reverse-complement of eGFP and the threshold value of 0.5. Reads where the normalized alignment score of the reverse-complement of eGFP was higher than the forward eGFP sequence and passed the threshold value of 0.5 were considered to be poly(T)-containing reads. For Barcode demultiplexing, first the sequence preceding the identified eGFP start was queried for the presence of the experiment-specific PCR front primer in the case of poly(A) reads, or its reverse-complement for poly(T) reads (sequences in Table 5). Next, the sequence between front primer and eGFP locations were used for barcode identification as described above.

### Comparing poly(A) and poly(A)/(T) end coordinates to eGFP sequence alignments

All alignments were performed using Smith–Waterman local alignments with Biostrings (Pages et al. 2019) (match score 1; mismatch score −1; gap opening penalty 0; and gap extension penalty 1). The normalized alignment score was calculated by dividing the local alignment score by the length of the query sequence. If not otherwise mentioned, alignments with a normalized alignment score below 0.6 were discarded as unspecific.

RNA reads with known poly(A) length were screened for the presence of the eGFP end sequence by querying the reverse FASTA string against the reverse eGFP end sequence (see Table 5). Reversing the FASTA sequence is necessary to achieve similar orientation between the raw signal in events tables (3′ to 5′) and FASTA string (initially 5′ to 3′). If the first three bases of the alignment are a perfect match, the sample index corresponding to the first alignment base is extracted by matching the number of the alignment character in the reversed FASTA string with the cumulative move count in the corresponding FAST5 file. This sample index is further used to compare with the sample index defined by *tailfindr* as representing the poly(A) end.

DNA reads were split based on the read type [poly(A)- or poly(T)-containing, see above]. Poly(A)-containing reads were treated similar to RNA reads (see above). Poly(T)-containing reads were screened for the presence of the eGFP end sequence by querying the original FASTA string against the reverse-complement of the reverse eGFP end sequence (see Table 5). The corresponding sample index is extracted as described for RNA reads (see above).

### *tailfindr* RNA poly(A) length estimation algorithm

To identify the signal corresponding to the poly(A) tails in RNA reads, the raw signal from ONT native RNA sequencing is extracted from the FAST5 files and *z*-normalized. Next, signal values above +3 and below −3 are truncated. The resulting processed raw signal is smoothened by a moving average filter (window size 400 samples; stride 1) to produce two smoothened signals: one by calculating the moving average from start to end, and one from end to start of the signal corresponding to the sequencing direction. Both smoothened signal vectors are then merged by point-by-point maximum calculation. Next, the calculated smoothened signal is segmented into regions being above or below 0.3. The expected signal of the ONT adapter consists of one segment above and one segment below 0.3 in the smoothened signal. The poly(A) tail immediately follows the Nanopore Adapter, thus the next segment in which the smoothened signal is above 0.3 is considered the poly(A) region, and the boundaries of this segment are considered the rough start and end of poly(A) tail (Fig. 1B). The threshold was chosen as the expected normalized signal for regions of homopolymer A on average is 0.89 and even a raw signal with two standard deviations below would result in a normalized signal of 0.55 (calculations in Supplemental Rmarkdown file).

The rough start and end are refined by first calculating a mean signal of the processed raw data contained between these boundaries through a moving average filter (window size 25; stride 25). Next, the slope of this mean signal is calculated between each two consecutive points. The boundaries of the longest continuous stretch of low-slope values (confined within bounds of +0.3 and −0.3 of slope signal) between the rough poly(A) start and end boundaries are considered the precise boundaries (Fig. 1B). The resulting poly(A) tail measurement in sample points is then normalized by the read-specific nucleotide translocation rate. To calculate the nucleotide translocation rate, the number of sample points per move is extracted from the FAST5 events table of each individual read (a “move” in raw data describes a single-nucleotide translocation through the pore as detected by base-calling). If a move of two is detected, two entries with each half the number of sample points are recorded; a move of two corresponds to a nucleotide translocation not identified by the base-caller. From the resulting vector of sample points per single move, the geometric mean is computed and used for normalization of poly(A) tail length.

### *tailfindr* DNA poly(A)/(T) estimation algorithm

Unlike RNA, DNA is double-stranded. Thus, both poly(A) and poly(T) homopolymer stretches can occur. To determine the read orientation, the Nanopore-specific front and end primer sequences (sequences in Table 5) are aligned against the first 100 bases extracted from FAST5 files. A read is considered poly(T)-containing if the normalized alignment score of end primer sequence is greater than that of the front primer sequence, and above the threshold of 0.6. Conversely, a read is considered poly(A)-containing if the normalized alignment score of front primer sequence is greater than that of the end primer sequence, and above the threshold of 0.6. To ensure that the full poly(A) tail is present in raw data, the last 50 bases of poly(A)-containing reads are queried for the presence of the reverse-complement end primer sequence. Reads where the normalized alignment score of the reverse-complement end primer is below 0.6 are considered truncated poly(A) reads and not analyzed further.

To identify borders of poly(A) or poly(T) stretches by similar procedures, the raw data of poly(A)-containing reads is reversed. Thus, both the poly(A) and poly(T) stretches are expected to be at the beginning of the raw signal. The alignment of end primer is considered the approximate start of the poly(A) or poly(T) stretch. Next, the raw data is z-normalized and converted to absolute values. To reduce computational workload, calculations to identify precise borders of poly(A)/(T) stretches are restricted to 3000 raw samples downstream from the rough poly(A)/(T) start site. This 3000-samples wide search window is wide enough to accommodate poly(A)/(T) tails of ∼350 nt length. The mean signal is generated by applying a sliding window (window size 10; stride 10) to the processed raw signal. Next, the slope of this mean signal is calculated between every two consecutive points. The precise start of the respective tail is considered to be the first location after the rough start site where the calculated slope is between −0.2 and 0.2, and the mean signal is between 0 and 0.3 for poly(T) reads and 0 to 0.6 for poly(A) reads. These thresholds contain all signal with two standard deviations away from the expected signal from homopolymer poly(T) or poly(A) stretches (calculation in Supplemental Rmarkdown file). To identify the precise tail end, the slope and the mean signals downstream from the precise tail start site are tested for violating their respective thresholds (see above). Since short non-tail-like signal spikes can randomly occur, we test the signal downstream from this tentative tail end for tail-like signal within thresholds until we either reach the end of the search window of 3000 sample points, or find another stretch of tail-like signal of at least 60 sample points in length. In the latter case, the tentative tail end is updated to the downstream tail end to account for the spike signal. The maximum allowable signal length exceeding the threshold that is located between two tail-like signals has been set to 120 nt (e.g., 120× read-specific nucleotide translocation rate).

The difference of the precise boundaries define the raw length of poly(A)/(T) stretches in sample points. This value is normalized by the read-specific nucleotide translocation rate calculated dependent on the respective base-calling strategy. For DNA reads base-called with standard models, the nucleotide translocation rate is defined as the geometric mean of the sample points per single move, as described for RNA poly(A) estimation. For flip-flop model base-calling, the raw signal is likely over-segmented resulting in too many nucleotide translocations (see Supplemental Discussion). To account for this, the average translocation rate is defined as the arithmetic mean of sample points per detected move after discarding the 5% highest outliers.

## SUPPLEMENTAL MATERIAL

Supplemental material is available for this article.

## DATA DEPOSITION

The code repository can be found at https://github.com/adnaniazi/tailfindr. The data repository can be found at https://www.ebi.ac.uk/ena/data/view/PRJEB31806.

## Supplementary Material

Supplemental Material
