# Supplementary material for: tailfindr: alignment-free poly(A) length measurement for Oxford Nanopore RNA and DNA sequencing
Source: RNA. 2019 Oct;25(10):1229–41. doi: 10.1261/rna.071332.119 (PMC6800471; doi:10.1261/rna.071332.119)
Supplement: Supplemental Material [file supp_25_10_1229__index.html]

tailfindr: Alignment-free poly(A) length measurement for Oxford Nanopore RNA and DNA sequencing — tailfindr: alignment-free poly(A) length measurement for Oxford Nanopore RNA and DNA sequencing — Supplemental Material 

# *tailfindr*: alignment-free poly(A) length measurement for Oxford Nanopore RNA and DNA sequencing

## Supplemental Material

- Supplemental\_FigS1.pdf
- Supplemental\_FigS2.pdf
- Supplemental\_FigS3.pdf
- Supplemental\_FigS4.pdf
- Supplemental\_FigS5.pdf
- Supplemental\_FigS6.pdf
- Supplemental\_FigS7.pdf
- Supplemental\_FigS8.pdf
- Supplemental\_Material.docx
