# Supplementary figures and images for: tailfindr: alignment-free poly(A) length measurement for Oxford Nanopore RNA and DNA sequencing
Source: RNA. 2019 Oct;25(10):1229–41. doi: 10.1261/rna.071332.119 (PMC6800471; doi:10.1261/rna.071332.119)

**A**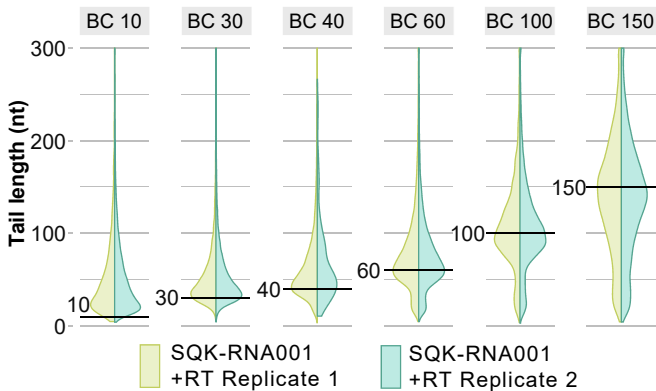**B**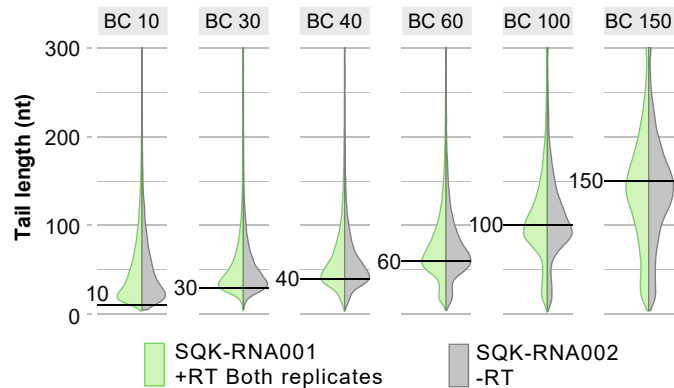**C**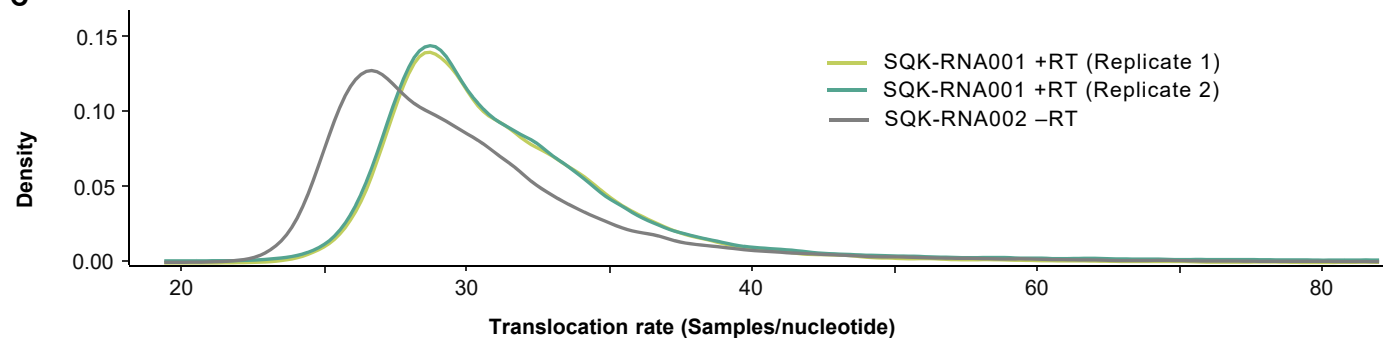

Supplement: Supplemental Material [file supp_071332.119_Supplemental_FigS1.pdf]

**A**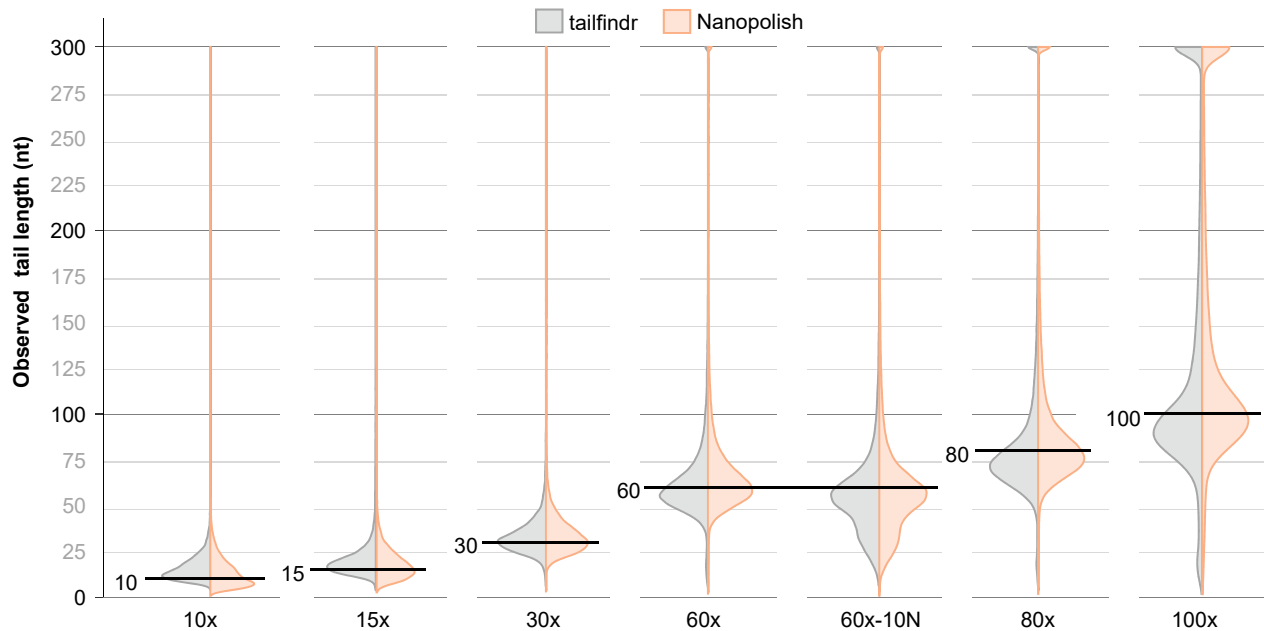**B**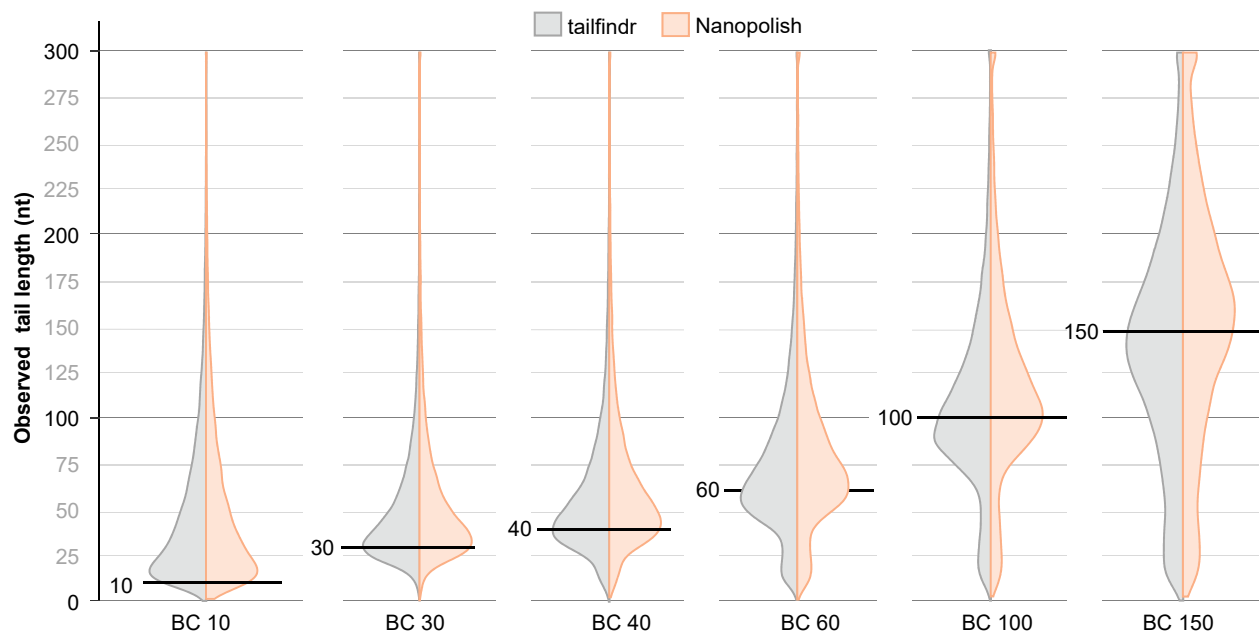

Supplement: Supplemental Material [file supp_071332.119_Supplemental_FigS2.pdf]

**A**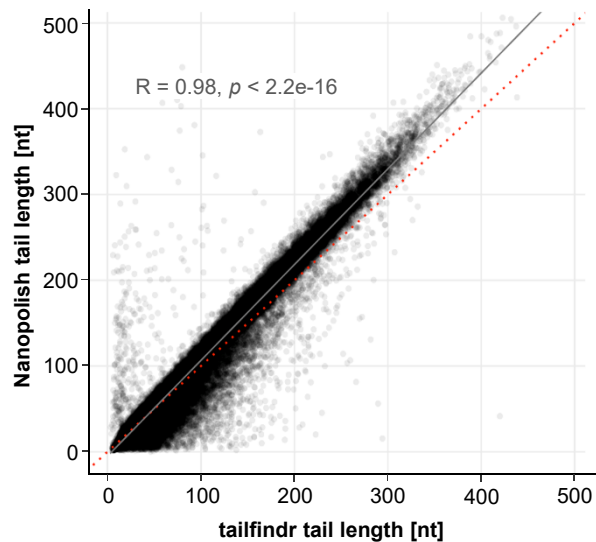**B**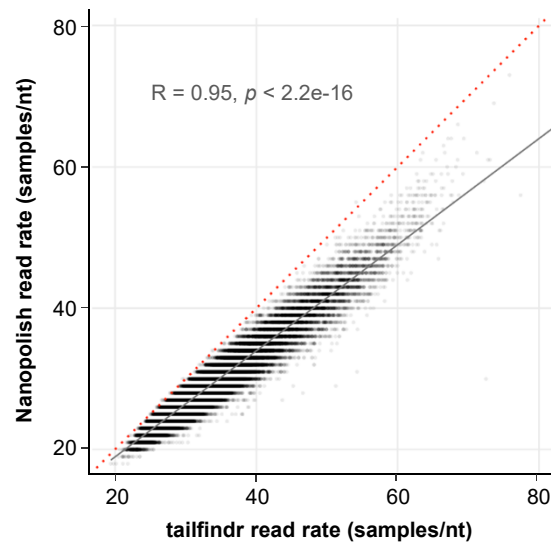**C**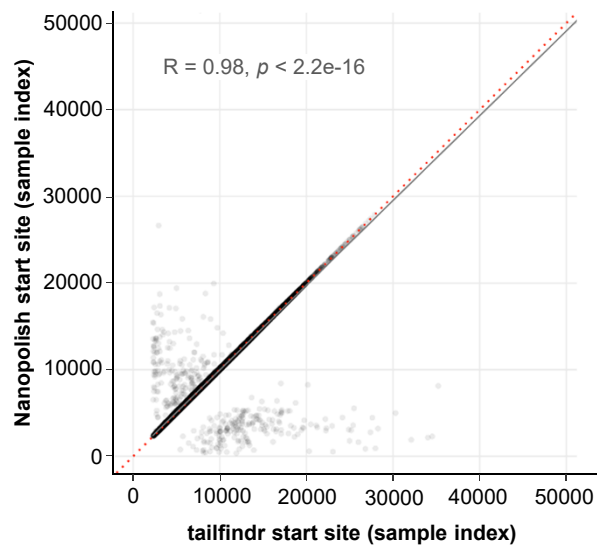**D**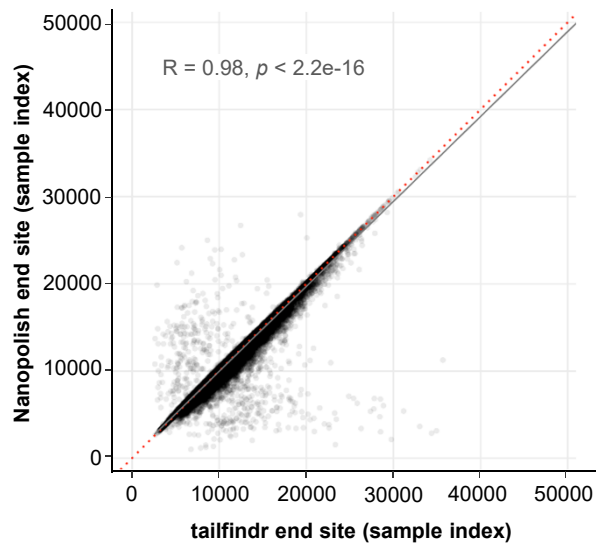

Supplement: Supplemental Material [file supp_071332.119_Supplemental_FigS3.pdf]

**A**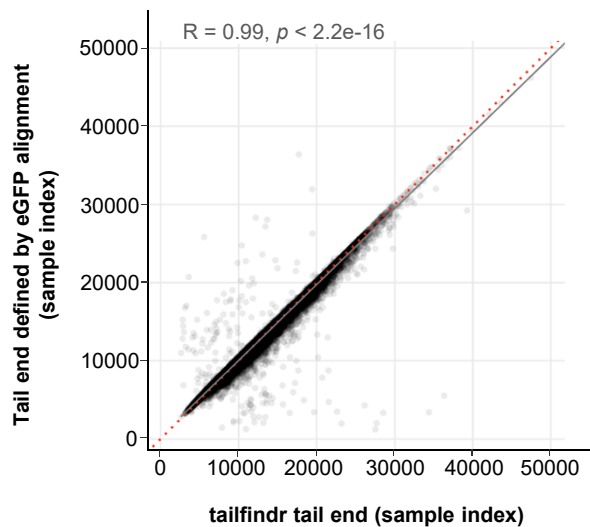**B**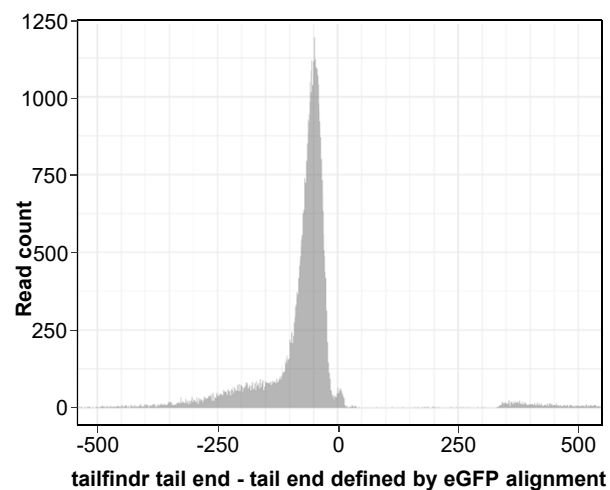**C**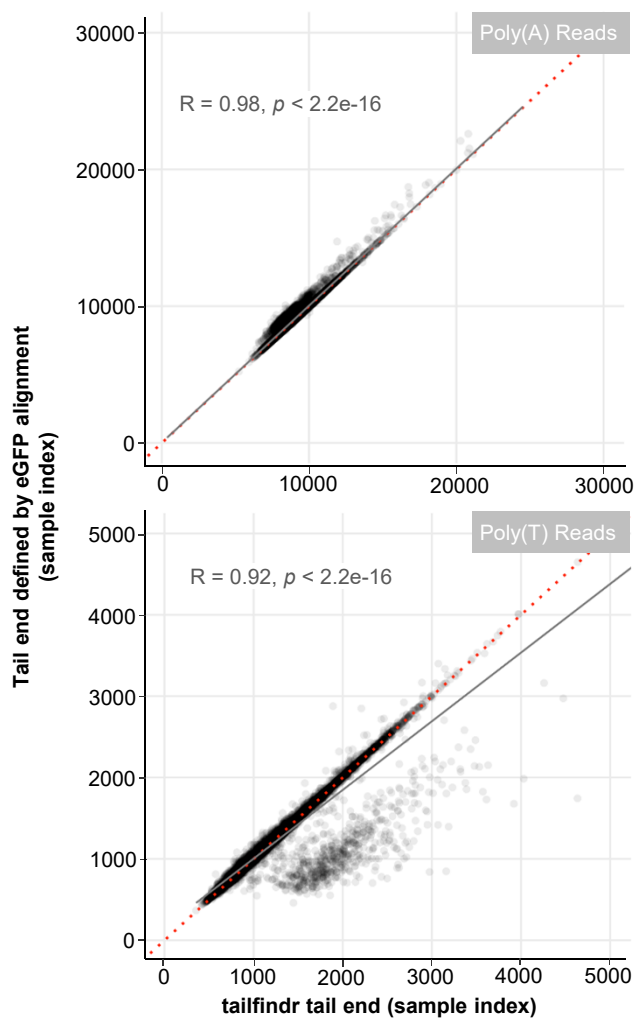**D**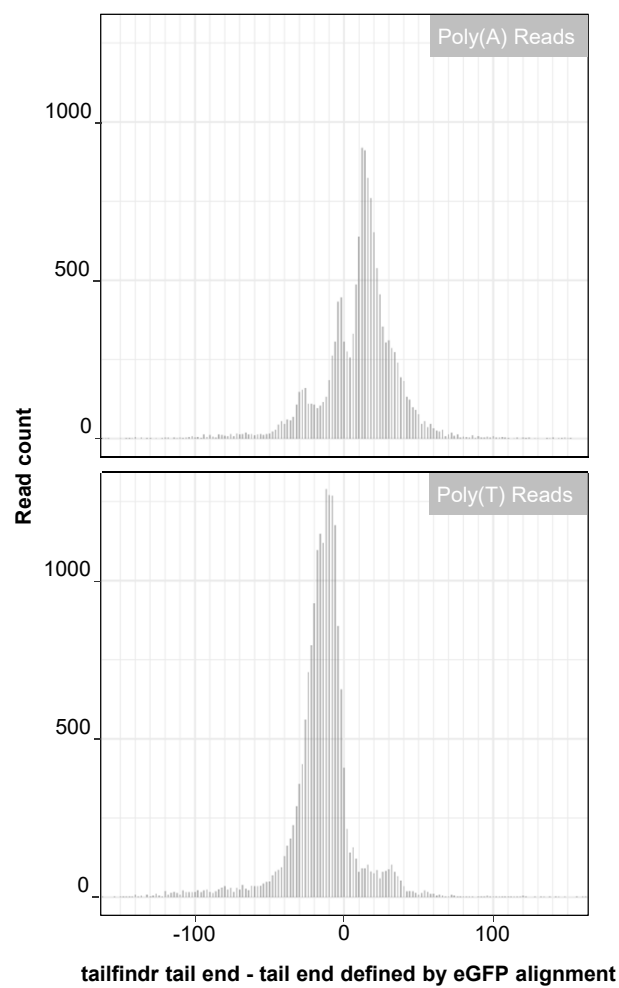

Supplement: Supplemental Material [file supp_071332.119_Supplemental_FigS6.pdf]

SQK-LSK 108

SQK-LSK 109

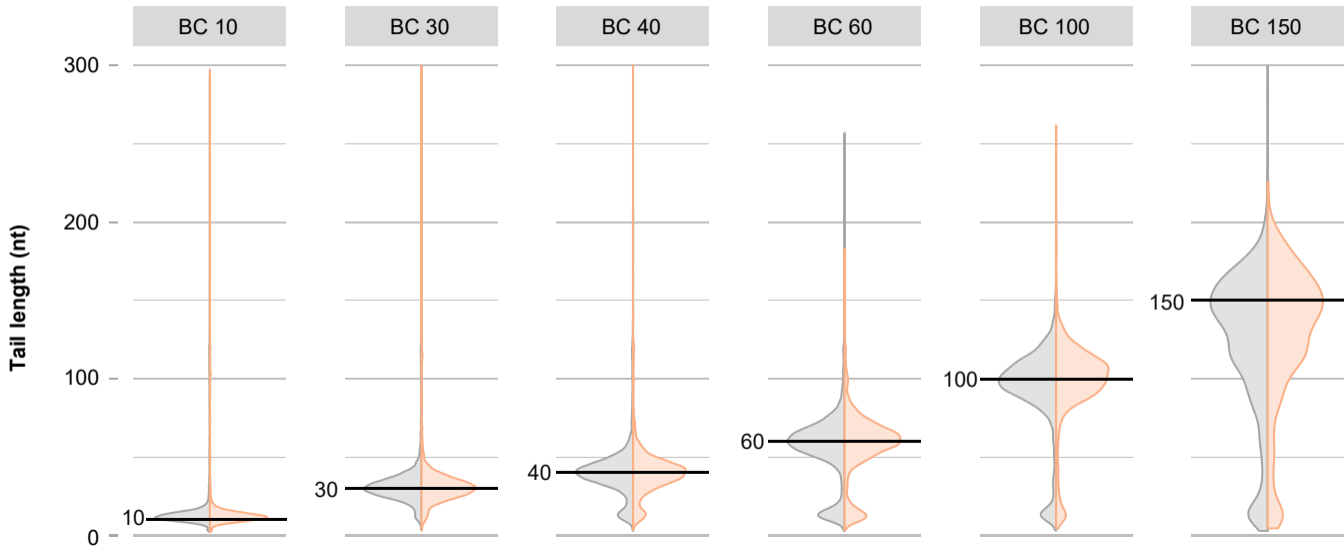

Supplement: Supplemental Material [file supp_071332.119_Supplemental_FigS7.pdf]

**A**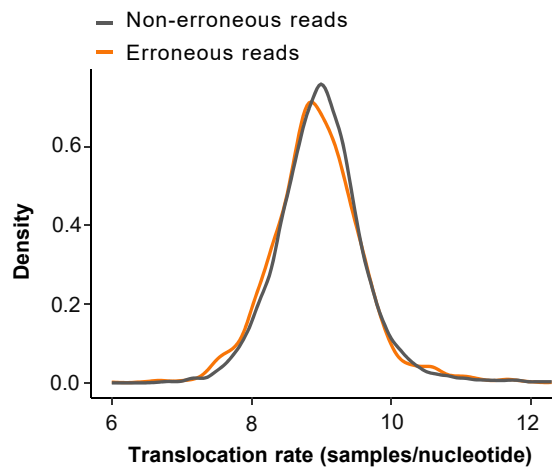**B**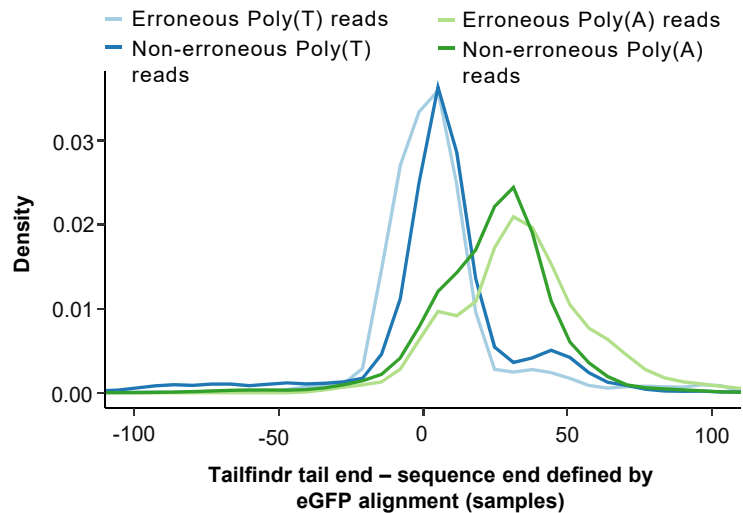**C**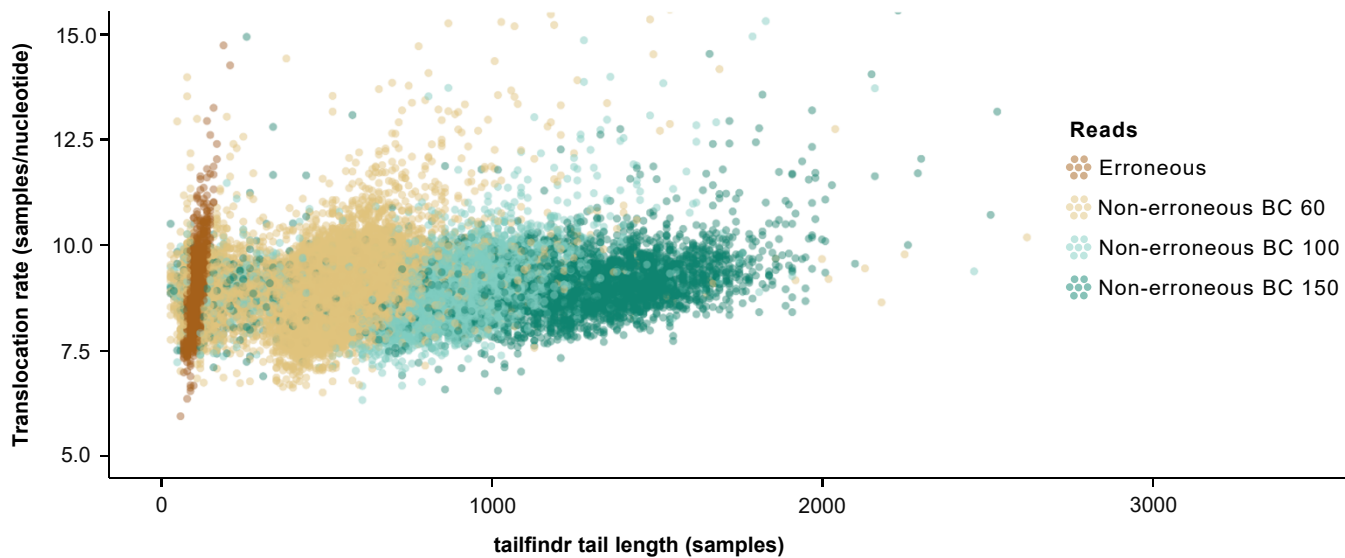

Supplement: Supplemental Material [file supp_071332.119_Supplemental_FigS8.pdf]
