# Supplementary material for: tailfindr: alignment-free poly(A) length measurement for Oxford Nanopore RNA and DNA sequencing
Source: RNA. 2019 Oct;25(10):1229–41. doi: 10.1261/rna.071332.119 (PMC6800471; doi:10.1261/rna.071332.119)

A

read_id	read_type	tail_is_valid	tail_start	tail_end	samples_per_nt	tail_length	file_path
cc4daa24-7875-427e-9aae-82fbacfc4052	invalid	FALSE	NA	NA	8.00	NA	/export/valenfs/data/processed_data
cc9281a2-cfe7-437f-ad29-2ecff087174c	invalid	FALSE	NA	NA	8.00	NA	/export/valenfs/data/processed_data
f41f79b3-e8e2-4e92-8253-a1d6bb6a8241	polyA	TRUE	7787	8007	8.01	27.48	/export/valenfs/data/processed_data
36b16025-2296-47d9-aee1-6041e39a5dc1	polyT	TRUE	572	1492	8.01	114.90	/export/valenfs/data/processed_data
fe305589-7070-42af-81b6-b1793fcd6a10	polyA	FALSE	NA	NA	8.01	NA	/export/valenfs/data/processed_data
30884743-6453-46be-8cd1-17449d21d430	polyA	FALSE	NA	NA	8.01	NA	/export/valenfs/data/processed_data
c0c2e47b-5931-4a9d-9a04-8f5f23dda00a	polyT	TRUE	983	1233	8.02	31.16	/export/valenfs/data/processed_data
1879fca0-f395-4d5e-92f0-e5d35d302183	polyT	TRUE	944	1454	8.02	63.55	/export/valenfs/data/processed_data
23f0d75a-6b24-4512-88b8-6f9661f23a45	polyA	FALSE	NA	NA	8.02	NA	/export/valenfs/data/processed_data
e0d3cb1e-fbaa-43e6-94a9-6110cd74c677	invalid	FALSE	NA	NA	8.02	NA	/export/valenfs/data/processed_data
7f959c69-a2db-4e86-996d-ee6064bdecb0	invalid	FALSE	NA	NA	8.02	NA	/export/valenfs/data/processed_data
7955afb7-ef49-49b6-8e88-cc23722a8a88	polyA	TRUE	7766	7896	8.03	16.18	/export/valenfs/data/processed_data
e6c5f92d-1b59-460f-a26c-b71bd5835965	polyT	TRUE	983	1193	8.03	26.15	/export/valenfs/data/processed_data

B

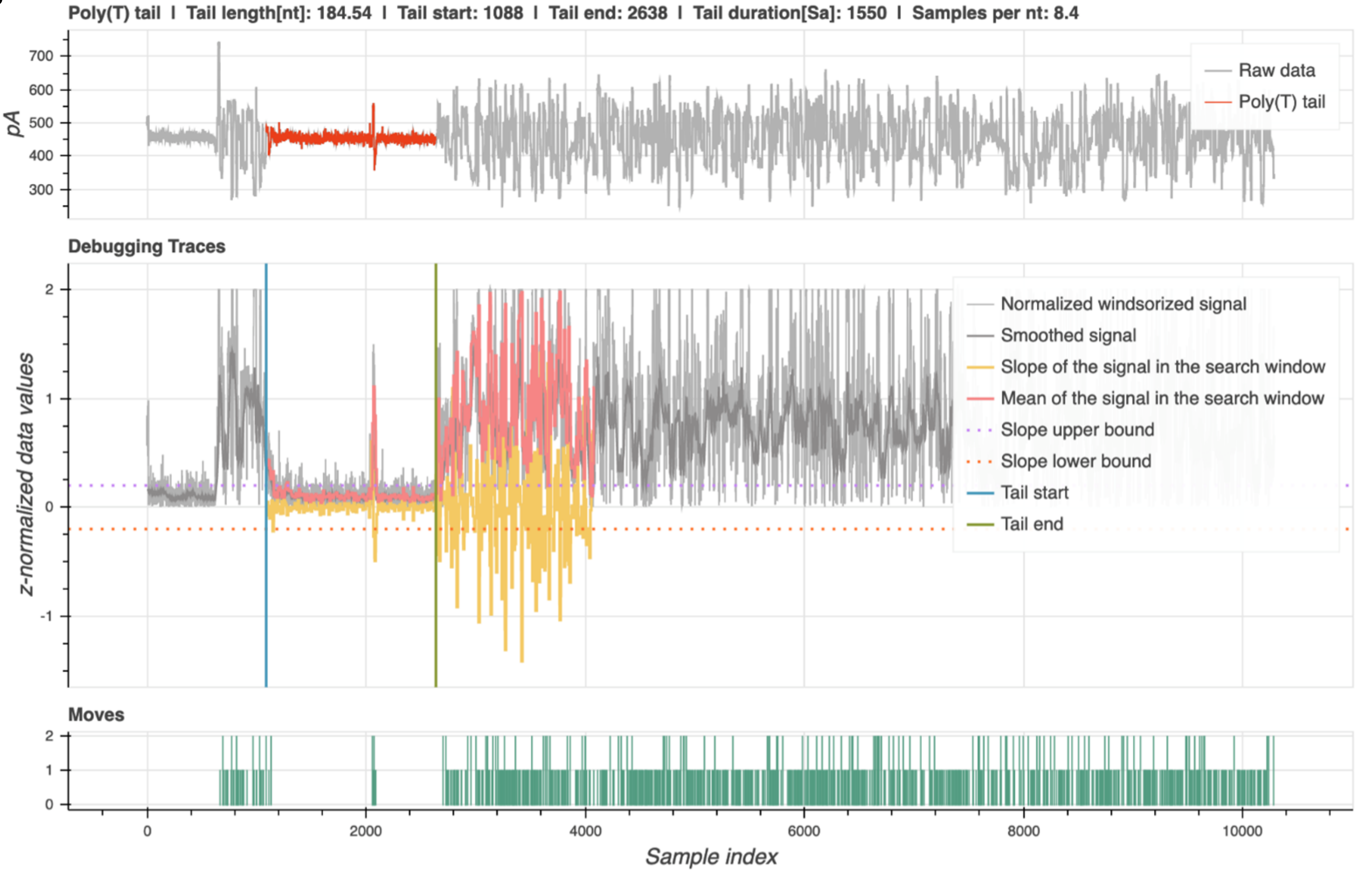

Supplement: Supplemental Material [file supp_071332.119_Supplemental_FigS5.pdf]
