# Supplementary material for: tailfindr: alignment-free poly(A) length measurement for Oxford Nanopore RNA and DNA sequencing
Source: RNA. 2019 Oct;25(10):1229–41. doi: 10.1261/rna.071332.119 (PMC6800471; doi:10.1261/rna.071332.119)
Supplement: Supplemental Material [file supp_071332.119_Supplemental_Material.docx]

**Supplementary Material to manuscript:**

***tailfindr*: Alignment-free poly(A) length measurement for Oxford Nanopore RNA and DNA sequencing**

# Supplementary Discussion

## Calculating the read-specific nuclear translocation rate

During Oxford Nanopore Sequencing, the translocation speed of the DNA or RNA molecule through the pore is not homogenous. Thus, the translocation rate for each individual nucleotide can differ significantly. The translocation speed can be influenced by the sequencing context [(Rang et al., 2018)](https://paperpile.com/c/FkxAv8/TCDn), but also by sample time and sequencing buffer conditions that change over the course of sequencing (unpublished communications). Furthermore, the translocation speed can be influenced by RNA or DNA modifications, which however should not affect RNA sequencing from *in vitro* transcribed molecules, or DNA sequencing of PCR-amplified molecules. Importantly, the motor protein for RNA and DNA differs, leading to dramatically different average translocation speed (70 nt for RNA in ONT Kit SQK-RNA001 vs. 450 nt for DNA in ONT Kit SQK-LSK108). In conclusion it is important to estimate the average nucleotide translocation rate for each read separately to account for the specific conditions at which the read was recorded.

During basecalling by ONT software, the raw data is averaged over multiple sample points. The resulting smoothened data is presented as ‘events’ in the ‘event table’ (newer basecalling by Guppy results in a ‘move table’). How many sample points are averaged depends on the sequencing and basecalling approach. Standard model basecalling for DNA uses 5 sample points for averaging, basecalling for RNA uses 15 sample points. These values cannot be changed by the user and are most likely chosen to sufficiently smoothen the raw data while minimizing the chance of missing a nucleotide translocation, as this roughly reflects half of sample points needed for an average nucleotide translocation (8 for DNA and 28 for RNA, own observations and unpublished communications). The basecaller then uses this smoothened data to identify nucleotide translocation based on signal level changes and the associated basepair context assigned to it. The resulting translocation is reported as ‘move’ in the ‘event table’. If a nucleotide translocation is missed based on signal level changes but could be identified by the basepair context, a move of 2 (equals to a translocation of 2 nucleotides at that point) is introduced. This strategy is prone to underestimate the number of nucleotide translocation, as it I) can miss translocations if they happen within the averaging window of 5 (DNA) or 15 (RNA) sample points, and II) because it does not sufficiently identify nucleotide translocations of homopolymers longer than five nucleotides. Additionally, slower nucleotide translocation due to sequence context, nucleotide modifications, translocation stalling or insufficient buffer conditions can lead to one-sided outliers of translocation rates. In sum, the basecalling strategy is prone to miss fast translocation and technical artefacts produce predominantly ‘slow’ translocations. This results in a nucleotide translocation rate distribution that is asymmetric towards higher values. We agree with Workman et al that this distribution follows a gamma distribution [(Workman et al., 2018)](https://paperpile.com/c/FkxAv8/FgDC). As long as no removal of outliers is performed, averaging the nucleotide translocation rate by the arithmetic mean of this distribution significantly overestimates the most common translocation rate (the mode of the distribution). Workman et al. address this problem by removing 5% of the most extreme outliers and calculating the median of the read-specific nucleotide translocations. However, while this approach produce length estimates in the range of expected poly(A) for RNA sequencing, it overestimates poly(A) tail lengths in DNA sequencing applications (data not shown). We therefore used the geometric mean of the read-specific single nucleotide translocations without discarding data points to calculate an average nucleotide translocation rate. This strategy results in robust estimation of poly(A) tail length for both ONT RNA and DNA sequencing approaches with standard model basecalling (Fig. 1D; Fig. 2C). Compared to Nanopolish polyA, this normalisation strategy results in slightly lower poly(A) length estimates, while the detected poly(A) boundaries were similar for both of the tools (Fig. S2; [Workman et al., 2018)](https://paperpile.com/c/FkxAv8/FgDC).

Oxford Nanopore Technologies recently presented an updated basecalling strategy with Flip-flop models instead of standard models used for neural network nucleotide decoding [(Oxford Nanopore Technologies, 2018b)](https://paperpile.com/c/FkxAv8/5PMZ). In this approach, the raw current level data is decoded by averaging 2 sample points instead of 5, resulting in higher-resolution basecalling. This strategy should increase the fidelity of basecalling on homopolymers as well as decrease the occurrence of missed nucleotide translocations. Indeed, we could not observe any move of 2 in resulting basecalled FAST5 files, which in most cases represent missed nucleotide translocations.

When calculating the read-specific nucleotide translocation rate from reads basecalled with standard and Flip-flop models, we routinely observe lower average values for Flip-flop model basecalling. Compared to standard model basecalling it is possible that Flip-flop model basecalling over-segments the raw data, resulting in too low average nucleotide translocation rates when calculated by the geometric mean. This is exemplified by the observation that the most frequently observed nucleotide translocation rate is 2 samples, the lowest possible value, and much lower than the so far assumed average nucleotide translocation rate of 8 (unpublished communications). To account for possible under-estimation of translocation rates, we therefore used the arithmetic mean of read-specific nucleotide translocation rates after discarding the 5% highest outliers as an approximation of the right normaliser, an approach used by Nanopolish polyA. Based on comparisons to results obtained by *tailfindr* after standard model basecalling on the same PCR-DNA molecules, this normalisation strategy results in correct poly(A) tail estimation for Flip-flop model basecalling.

## Bimodal distribution in DNA poly(A) estimation

By applying *tailfindr* on PCR-amplified DNA-sequences with known poly(A) length we showed that poly(A) and poly(T) stretches overall match the expected poly(A)/(T) length (Figure 2). However, for longer poly(A)/(T) stretches, a second minor population of poly(A)/(T) length of around 12 nt could be observed that is not present in RNA sequencing approaches where the RNA was *in vitro* transcribed from the same PCR samples (Figure 2C, D).

While we are unsure about the origin of the secondary population, we can exclude that these measurements result from erroneous poly(A)/(T) estimation by the *tailfindr* algorithm. Analysing these reads separately has shown that the average nucleotide translocation speed used to calculate the poly(A)/(T) length is similar to the translocation speed observed on non-erroneous reads (Fig. S8A,C). Additionally we could show that the poly(A)/(T) length in raw data roughly corresponds to a poly(A)/(T) length in nucleotides as calculated (Fig. S8C). Querying the sequence directly adjacent to the identified poly(A)/(T) borders further verified that the following sequence is indeed the expected eGFP coding sequence (Fig S8B). Here, poly(A) and poly(T) containing reads show different trends because of their orientation. In both cases, the distance between poly(A)/(T) tail end and eGFP sequence alignment coordinate is within 3 nucleotides, as on average around 10 sample points correspond to one nucleotide translocation. The PCR-DNA molecules sequenced here has gone through several rounds of size-selection based on Agarose gel extraction. We can thus also exclude the possibility that a population of shorter-than-expected poly(A)/(T) tails is produced during PCR barcoding for molecule identification.

One possible explanation for the observed second poly(A)/(T) length population could be that during Library preparation the poly(A)/(T) stretch was partially degraded. This could for example happen during the essential dA-tailing reaction, where single-stranded DNA ends are either enzymatically removed or filled to result in a 3’ dA overhang necessary for Adapter ligation. Another possibility is that during DNA ligation incubation, intermolecular ligation occurred. This could result in combination of a 5’ barcode with a 3’ poly(A)/(T) section other than the expected length. Frequently observed molecules of longer length than the expected 1kb PCR product show that these chimeric reads can occur. However, we accounted for this possibility by filtering out reads that are longer or shorter than the expected PCR molecule length. Another possibility is that during PCR amplification, a wrong primer encoding a 5’ barcode other than expected was paired with the poly(A)-containing 3’ primer, leading to molecules with the wrong barcode attached. However, since several rounds of nested PCR were performed it is unlikely to result in a significant amount of wrong PCR products of the same length across several samples. In conclusion, while we could rule out biases in our poly(A) length algorithm, we cannot rule out that the used PCR samples contain shorter than expected poly(A)/(T) tails by imprecision during sample preparations.

## Comparing poly(A)/(T) estimation across replicates with different Library preparation kits

*tailfindr* uses a read-specific nucleotide translocation rate to estimate the poly(A) length in basepairs from raw data (see Methods and Supplementary discussion). This parameter can be affected by sequencing context [(Rang et al., 2018)](https://paperpile.com/c/FkxAv8/TCDn), sample time and sequencing buffer conditions, among others (unpublished communications). It is therefore possible that poly(A) tail estimates are not robust over different library preparation experiments. In this study, we replicated RNA sequencing of *in vitro* transcribed eGFP RNA on two occasions with the same Library preparation kit. As can be seen in Supplementary Figure S1A, poly(A) length estimates are almost identical between replicates. We further repeated the experiment with the recently released SQK-RNA002 Library preparation kit. Again, obtained results are almost identical across all screened barcodes (Figure S1B), even though the read-specific nucleotide translocation rate is much lower for data from this new kit (Fig. S1C). Similarly, for DNA sequencing experiments performed with two different Library preparation kits (SQK-LSK108 and SQK-LSK109), poly(A)/(T) length estimation results are very similar across all measured poly(A)/(T) tail lengths (Fig. S7). We thus conclude that poly(A) tail measurements using ONT sequencing is reproducible, and that changes in translocation speed from sequencing chemistry changes can be accounted for by *tailfindr* poly(A) length estimation.

## Validating poly(A) end boundary by sequence alignment

Poly(A) and poly(T) segment definition by *tailfindr* is purely based on raw data. To validate that the identified boundary corresponds to the beginning of the RNA or DNA sequence, we compared the coordinates of the poly(A)/(T) end as defined by *tailfindr* with the coordinates corresponding to the expected eGFP sequence adjacent to the poly(A) tail (see Methods for details). Sample index coordinates of sequence alignment is highly correlated with the identified poly(A) tail end in RNA (R = 0.99, p < 2.2e-16; Fig. S6A). The difference between the defined poly(A) tail end coordinate and eGFP alignment coordinate for most reads is around 50 sample points (Fig. S6B), which corresponds to around 2 nucleotides when assuming an average nucleotide translocation rate of 35. The histogram of this difference shows mainly negative values, which could be explained by basecaller uncertainty in defining the boundary between basepair transitions close to homopolymer stretches, which is a known problem [(Rang et al., 2018)](https://paperpile.com/c/FkxAv8/TCDn). This is further exemplified when analysing DNA sequencing data in a similar manner (Fig. S6C + D). Again, correlation between poly(A) or poly(T) end coordinates and sequence alignment coordinates is very strong (R= 0.98 and 0.92, respectively; p < 2.2e-16 for both). While the difference of the two measures for poly(T) reads is again negative and centers at 10 sample points (corresponds to 1 nucleotide difference), poly(A) containing reads show mainly positive values (Fig. S6D). This is a consequence of the different orientation of the read, where first the main sequence is queried before the poly(A) tail region is recorded in raw data. Positive values in this diagram suggest that sequence alignment coordinates are smaller than poly(A) end coordinates, which again could be explained by low precision of the basecaller in defining the boundary between nucleotide transitions at the border towards homopolymer stretches. As discussed for poly(T) containing reads above, the difference is mainly explained by the location; poly(A) tails are at the end of sequenced molecules, and thus the measurement is prone to inaccuracies resulting from deviating signal in the main sequence. Regardless, most reads show a difference corresponding to 3 nucleotides, which is low compared to the overall variation observed in poly(A) tail estimation (Fig. 2C).

In conclusion, *tailfindr* correctly identifies poly(A) tail boundaries and boundary detection does not significantly contribute to the variability of poly(A) length measurements.

#

# Supplementary Data links

Code repository: <https://github.com/adnaniazi/tailfindr>

Rmarkdown analysis repository: <https://github.com/adnaniazi/krauseNiazi2019Analyses>

Data repository: <https://www.ebi.ac.uk/ena/data/view/PRJEB31806>

#

# Supplementary Figure Legends

**Figure S1 | Poly(A) length estimates are robust over library preparation differences**

**S1A** Vertical density plots comparing two biological replicates (replicate 1, green; replicate 2, turquoise) of *in vitro* transcribed eGFP-RNA molecules with known poly(A) tail length (from left to right: 10 nt, 30 nt, 40 nt, 60 nt, 100 nt, 150 nt labelled as BC10, BC30, BC40, BC60, BC100 and BC150, respectively). Horizontal black lines demarcate expected poly(A) length for individual barcodes. Poly(A) estimates exceeding 300 nt were set to 300 prior to plotting.

**S1B** Vertical density plots comparing data generated with Library preparation kit SQK-RNA001 with reverse transcription (green) with data generated with Library preparation kit SQK-RNA002 without reverse transcription (grey). Samples and plotting conditions as above.

**S1C** Density plots comparing the read-specific nucleotide translocation rate (x-axis) of RNA sequencing experiments performed either with the Library preparation kit SQK-RNA001 (replicate 1, green; replicate 2, turquoise) or with SQK-RNA002 without optional reverse transcription (grey).

**Figure S2 | Comparison of poly(A) estimation by *tailfindr* and Nanopolish polyA**

**S2A** Vertical density plots of poly(A) length estimation from *tailfindr* (grey) and Nanopolish polyA (orange) on data published by Workman et al., 2018. The recorded data contains *in vitro* transcribed yeast enolase RNA with known poly(A) lengths (from left to right: 10 nt, 15 nt, 30 nt, 60 nt, 60 nt poly(A) and 10 random nucleotides, 80 nt, 100nt). Poly(A) estimates exceeding 300 nt were set to 300 prior to plotting.

**S2B** Vertical density plots of poly(A) length estimation from *tailfindr* (grey) and Nanopolish polyA (orange) on *in vitro* transcribed eGFP-RNA with known poly(A) lengths (from left to right: 10 nt, 30 nt, 40 nt, 60 nt, 100 nt, 150 nt labelled as BC10, BC30, BC40, BC60, BC 100 and BC150, respectively). Poly(A) estimates exceeding 300 nt were set to 300 prior to plotting.

**Figure S3 | Comparison of poly(A) tail estimation from *tailfindr* and Nanopolish polyA**

**S3A** Scatter plot of poly(A) length estimation from *tailfindr* (x-axis) and Nanopolish polyA (y-axis) on *in vitro* transcribed eGFP-RNA with different poly(A) lengths. Grey line indicates Pearson-correlation fit, red dashed line indicates x=y. (R, p by Pearson correlation)

**S3B** Scatter plot of read-specific nucleotide translocation rate used to normalise poly(A) tail length from *tailfindr* (x-axis) and Nanopolish polyA (y-axis). Grey line indicates Pearson-correlation fit, red dashed line indicates x=y. (R, p by Pearson correlation)

**S3C** Scatter plot of identified poly(A) start from *tailfindr* (x-axis) and Nanopolish polyA (y-axis). Grey line indicates Pearson-correlation fit, red dashed line indicates x=y. (R, p by Pearson correlation)

**S3D** Scatter plot of identified poly(A) end from *tailfindr* (x-axis) and Nanopolish (y-axis). Grey line indicates Pearson-correlation fit, red dashed line indicates x=y. (R, p by Pearson correlation)

**Figure S4 | Example data output from *tailfindr* on RNA sequencing data**

**S4A** Tabular output containing the unique ONT read ID, tail start and end coordinates, the normaliser (samples_per_nt), the calculated tail length in nt (“tail_length”) and the filepath for each individual read.

**S4B** Example debugging plots that can be optionally generated by *tailfindr* for highlighting the poly(A) tail. Displayed is a poly(A) tail of *in vitro* transcribed eGFP RNA. Top panel shows the total raw data with highlighted poly(A) section (red). Middle panel shows calculated signal derivatives used to define the poly(A) tail boundaries for debugging purposes. Bottom panel displays detected moves as recorded by the basecaller (green).

**Figure S5 | Example data output from *tailfindr* on DNA sequencing data**

**S5A** Tabular output containing the unique ONT read ID, read orientation (“read_type”), tail start and end coordinates, the normaliser (samples_per_nt) and the calculated tail length in nt (“tail_length”), among others.

**S5B** Example debugging plots that can be optionally generated by *tailfindr* for highlighting the poly(A)/(T) sections. Displayed is a poly(T) stretch of eGFP PCR-DNA basecalled with standard model. Top panel shows the total raw data with highlighted poly(T) tail section (red). Middle panel shows calculated signal derivatives used to define the poly(T) tail boundaries for debugging purposes. Bottom panel displays detected moves as recorded by the basecaller (green). Important to notice is a small signal spike in the raw data that *tailfindr* allows to be included in the full poly(T) tail.

**Figure S6 | *tailfindr* poly(A) ends agree with sequence start as defined by alignment**

**S6A** Scatter plot of poly(A) end defined by *tailfindr* (x-axis) and sequence start defined by eGFP alignment (y-axis) on native RNA sequencing of *in vitro* transcribed eGFP RNA with known poly(A) tail lengths. Grey line indicates Pearson-correlation fit, red dashed line indicates x=y. (R, p by Pearson correlation)

**S6B** Histogram of the difference of poly(A) end and sequence start coordinates in sample points (x-axis). On average, 35 sample points correspond to one nucleotide translocation.

**S6C** Scatter plots of poly(A) (upper panel) and poly(T) (lower panel) ends defined by *tailfindr* (x-axis) and sequence start defined by eGFP alignment (y-axis) on DNA sequencing of PCR-amplified eGFP samples with known poly(A) tail lengths. Grey line indicates Pearson-correlation fit, red dashed line indicates x=y. (R, p by Pearson correlation)

**S6D** Histograms of the difference of poly(A) (upper panel) and poly(T) (lower panel) end and sequence start coordinates in sample points (x-axis). On average, 10 sample points correspond to one nucleotide translocation in DNA sequencing experiments..

**Figure S7 | *tailfindr* poly(A)/(T) estimation on DNA sequencing is robust over replicates**

Vertical density plot of poly(A)/(T) length estimates on two replicates of PCR-amplified eGFP coding sequence with known poly(A) lengths obtained with two different Library preparation kits (SQK-LSK108, grey; SQK-LSK109, orange). Horizontal black lines demarcate expected poly(A) length for individual barcodes (from left to right: 10 nt, 30 nt, 40 nt, 60 nt, 100 nt, 150 nt labelled as BC10, BC30, BC40 ,BC60, BC100 and BC150, respectively).

**Figure S8 | Short poly(A)/(T) length estimates do not result from erroneous poly(A)/(T) tail definition or normalisation**

**S8A** Density plot of nucleotide translocation rate (x-axis) used to normalise PCR-amplified eGFP samples with known poly(A) tail length for reads that show expected (grey) and too low (orange) poly(A)/(T) estimates.

**S8B** Density plot of the difference of poly(A)/(T) tail end and sequence alignment coordinates (x-axis) for poly(A) (green) or poly(T) (blue) reads either showing expected poly(A)/(T) length (dark color) or too low estimates (light color).

**S8C** Scatter plot of poly(A)/(T) length estimates (x-axis) and nucleotide translocation rate (y-axis) for PCR-amplified eGFP samples with poly(A)/(T) tails of 60 nt (sand), 100 nt (turquoise) and 150 nt (dark green) as well as reads that show too low poly(A)/(T) tail estimates (brown)
