# Supplementary material for: tailfindr: alignment-free poly(A) length measurement for Oxford Nanopore RNA and DNA sequencing
Source: RNA. 2019 Oct;25(10):1229–41. doi: 10.1261/rna.071332.119 (PMC6800471; doi:10.1261/rna.071332.119)

A

read_id	tail_start	tail_end	samples_per_nt	tail_length	file_path
56aae807-0f20-4e28-a5e2-5a9c2b1e1509	5701	8176	41.16	60.13	/export/valenfs/data/processed_data/MinION/20181...
56aa4686-d5f3-4d79-901d-736ad71f4aa5	9084	9884	57.25	13.97	/export/valenfs/data/processed_data/MinION/20181...
56ae532c-6199-4611-8e77-f7e4233f3431	10824	12324	48.79	30.75	/export/valenfs/data/processed_data/MinION/20181...
88f00dc8-607b-47b0-a9db-1123cca05934	9380	10755	55.17	24.92	/export/valenfs/data/processed_data/MinION/20181...
9b3ae14c-46b7-4577-abe3-01481cf4a457	8057	9232	48.57	24.19	/export/valenfs/data/processed_data/MinION/20181...
ab1d2036-5e4c-417b-8f48-1855b0246a23	6505	12005	41.23	133.41	/export/valenfs/data/processed_data/MinION/20181...
2c66c4e0-e314-42f4-81f4-94cbf8a4a6c3	8030	9905	48.85	38.38	/export/valenfs/data/processed_data/MinION/20181...
fbb47523-d1ad-45f2-9dab-c69c54082c0a	8726	9726	52.81	18.93	/export/valenfs/data/processed_data/MinION/20181...
11cc0eac-0410-4f8c-8c27-88a1c7492da3	9633	11258	44.85	36.23	/export/valenfs/data/processed_data/MinION/20181...

B

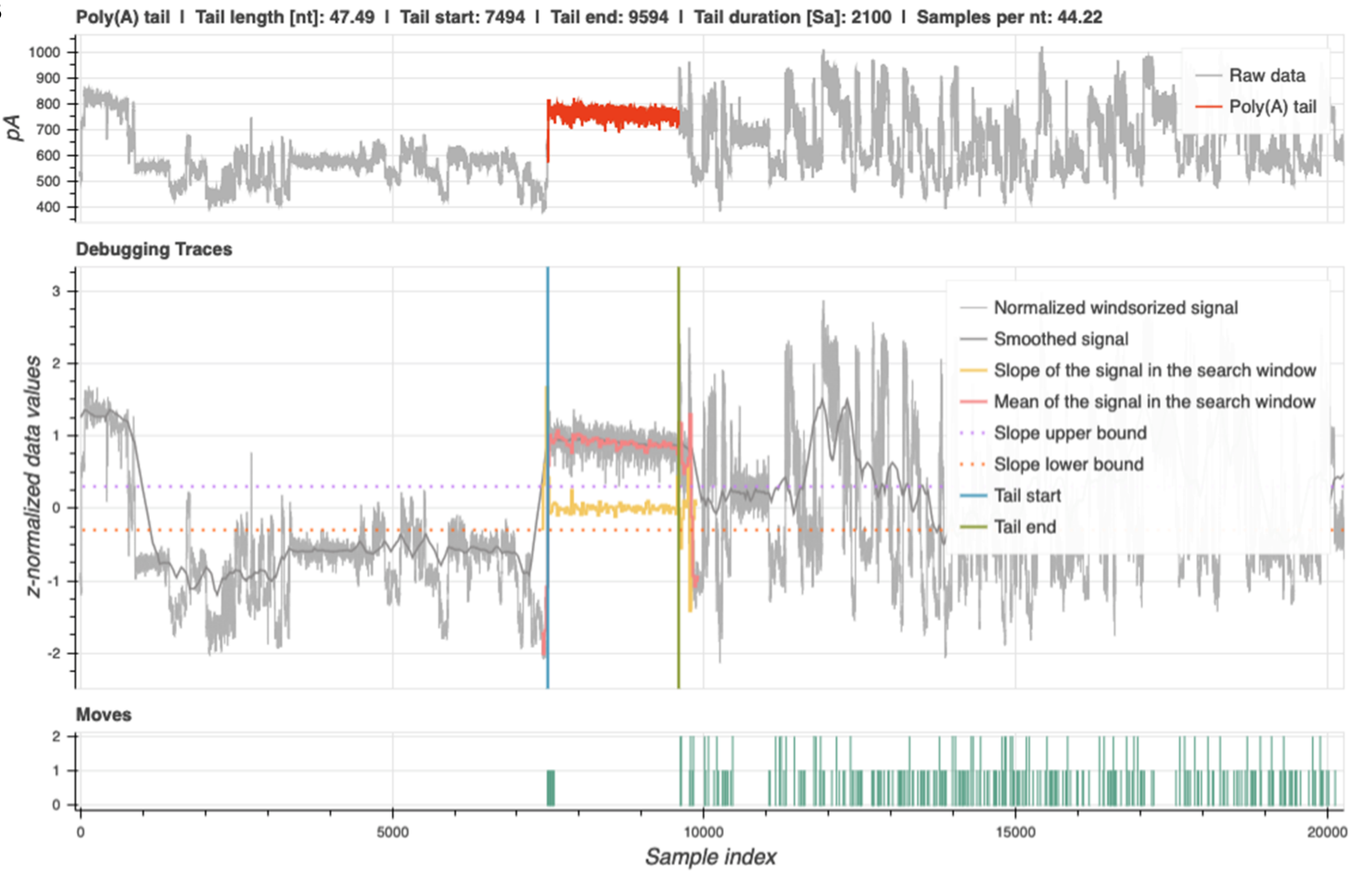

Supplement: Supplemental Material [file supp_071332.119_Supplemental_FigS4.pdf]
